# BNIP3-mediated mitophagy boosts the competitive growth of Lenvatinib-resistant cells via energy metabolism reprogramming in HCC

**DOI:** 10.1038/s41419-024-06870-9

**Published:** 2024-07-05

**Authors:** Sikai Wang, Hongxia Cheng, Miaomiao Li, Dongmei Gao, Haoran Wu, Shanshan Zhang, Yilan Huang, Kun Guo

**Affiliations:** 1 Liver Cancer Institute, Zhongshan Hospital, Key Laboratory of Carcinogenesis and Cancer Invasion, Ministry of Education, Fudan University, Shanghai, 200032 China; 2 Department of Radiation Oncology, Shanghai Pulmonary Hospital, Tongji University School of Medicine, Shanghai, 200032 China; 3 Endoscopy Center, Zhongshan Hospital, Fudan University, Shanghai, 200032 China; 4 Department of Anatomy, Histology and Embryology, School of Basic Medical Sciences, Shanghai Medical College, Fudan University, Shanghai, 200032 China; 5 Cancer Research Center, Institute of Biomedical Science, Fudan University, Shanghai, 200032 China

**Keywords:** Cancer metabolism, Mitophagy

## Abstract

An increasing evidence supports that cell competition, a vital selection and quality control mechanism in multicellular organisms, is involved in tumorigenesis and development; however, the mechanistic contributions to the association between cell competition and tumor drug resistance remain ill-defined. In our study, based on a contructed lenvitinib-resistant hepatocellular carcinoma (HCC) cells display obvious competitive growth dominance over sensitive cells through reprogramming energy metabolism. Mechanistically, the hyperactivation of BCL2 interacting protein3 (BNIP3) -mediated mitophagy in lenvatinib-resistant HCC cells promotes glycolytic flux via shifting energy production from mitochondrial oxidative phosphorylation to glycolysis, by regulating AMP-activated protein kinase (AMPK) -enolase 2 (ENO2) signaling, which perpetually maintaining lenvatinib-resistant HCC cells’ competitive advantage over sensitive HCC cells. Of note, BNIP3 inhibition significantly sensitized the anti-tumor efficacy of lenvatinib in HCC. Our findings emphasize a vital role for BNIP3-AMPK-ENO2 signaling in maintaining the competitive outcome of lenvitinib-resistant HCC cells via regulating energy metabolism reprogramming; meanwhile, this work recognizes BNIP3 as a promising target to overcome HCC drug resistance.

## Introduction

Liver cancer, more explicitly hepatocellular carcinoma (HCC), is the fourth primary cause of cancer-associated mortality and its incidence is constantly ascending worldwide [1]. At present, surgical treatments are the primary strategy for long-term survival in early-stage HCC patients [2, 3]. However, owing to the lack of typical clinical symptoms and rapid progression of HCC, over 70% of the patients are diagnosed with advanced HCC. As the FDA-approved first-line targeted agents for advanced unresectable HCC, sorafenib and lenvatinib display a favorable outcome; however, the inevitable adverse effects and low objective response rate suggest that drug resistance has become a major obstacle in the treatment of advanced HCC [4]. At present, despite progress in drug resistance reversal tactics including the log kill method, synthetic lethal strategy and immunotherapies based on biological roots such as tumor burden, growth kinetics and tumor microenvironment [5–7], there are many open questions and challenges.

As is well-known, tumor heterogeneity is widely recognized as a characteristic of enabling tumors to overcome evolutionary pressures [8, 9] and plays a critical role in the development of tumor drug resistance [10]. Recently, some evidence supports that cell competition, a vital selection and quality control mechanism in multicellular organisms, promotes phenotypic heterogeneity in tumors [11, 12]. For example, DNMT3A mutation endows hematopoietic stem cells (HSCs) with fitness advantage and prompts them to defeat unmutated HSCs after transplantation, leading to acute leukemia initiation [13]. Through forming a favorable microenvironment of crypt colonization, intestinal stem cells (ISCs) with Apc, Kras, or PIK3CA mutation as supercompetitors actively compete and remove normal ISCs, eventually driving colorectal cancer development [14, 15]. Of note, cells with MYC mutations have been shown to result competitively in the apoptosis of neighbored normal cells via increasing lactate availability [16], consistent with the metabolic features of drug-resistant cells. Therefore, there is a pressing need to understand how cell competition contributes to the development of drug resistance and leverage this knowledge to target cell competition-driven drug resistance in tumors.

In the current study, we have demonstrated for the first time that cell competition promotes drug resistance development in HCC by facilitating the dominant growth of lenvatinib-resistant HCC cells. Mechanistically, the hyperactivation of BNIP3-mediated mitophagy in lenvatinib-resistant HCC cells promotes glycolytic flux via shifting energy production from mitochondrial oxidative phosphorylation to glycolysis by regulating AMPK-ENO2 signaling, which perpetually maintains lenvatinib-resistant cells’ competitive dominance against sensitive HCC cells. More importantly, the combination of BNIP3 inhibition and lenvatinib significantly suppress resistance-driven HCC progression in vivo. Altogether, these results help us further understand the nature of cell competition-driven drug resistance and provide a promising approach to overcome drug resistance in HCC.

## Results

### Cell competition drives the dominant growth of lenvatinib-resistant cells in HCC

**Fig. 1:**
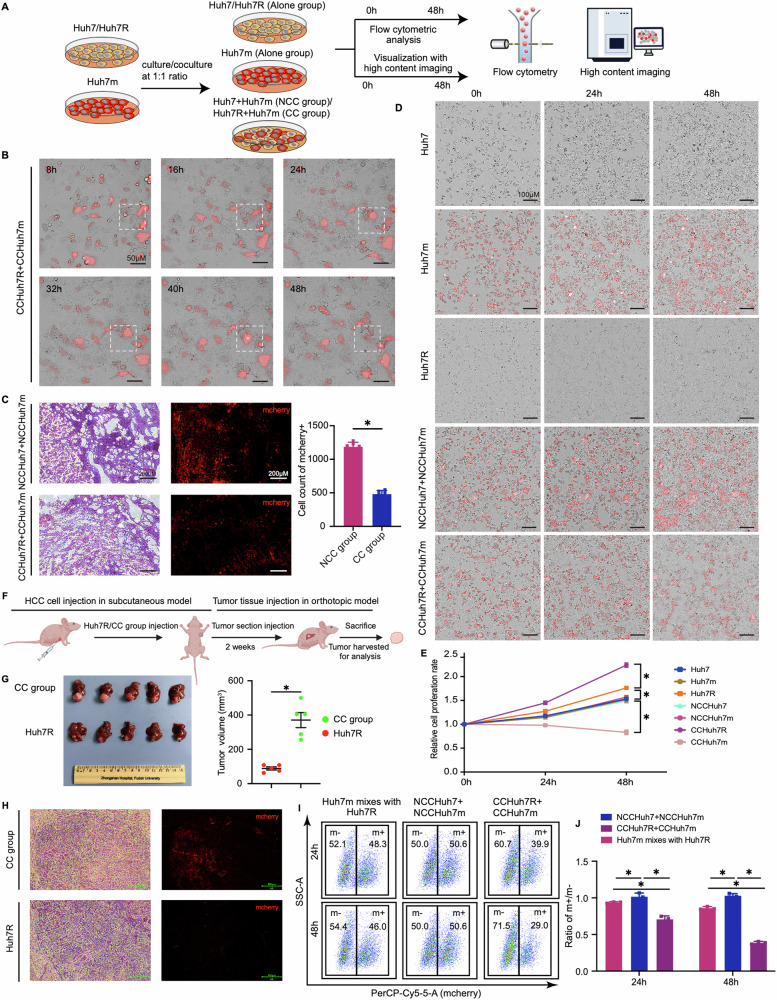
Cell competition drives the dominant growth of lenvatinib-resistant cells in HCC. **A** Schematic workflow of our experimental strategy. Huh7 or Huh7R was cocultured with Huh7m at 1:1 ratio or cultured alone respectively. Cells were cultured for 48 h after cell attachment and then performed by high content imaging analysis and flow cytometry analysis. **B** High content imaging of cell growth over a time course of coculture after cell attachment in CC group (CCHuh7R+CCHuh7m) using 20X objective lens (Details see Video 5). **C** (Left) Representative H&E stain and fluorescence imaging of cells in NCC group-and CC group-mice. (Right) Mcherry positive cells in NCC group and CC group were counted with ImageJ. *N* = 5. **D** High content imaging of cell growth at 0 h, 24 h, 48 h after cell attachment in Alone group (Huh7, Huh7m, Huh7R), NCC group (NCCHuh7+NCCHuh7m) and CC group (CCHuh7R+CCHuh7m) using 10X objective lens (Details see Video 6–10). **E** Quantitative statistics of cell proliferation rate in (**D**). **F**, **G** Schematic workflow (**F**), tumor photos and statistic analysis of tumor volume (**G**) in our HCC orthotopic model. *N* = 5. **H** (Left) Representative H&E stain and fluorescence imaging of cells in Huh7R group-and CC group-mice. (Right) Mcherry positive cells in CC group were counted with ImageJ. *N* = 5. **I** Flow cytometry analysis of cell proportion in NCC group, CC group and single Huh7m mixed with single Huh7R at 24 h and 48 h after cell attachment. mcherry+ (m+): Huh7m, NCCHuh7m, CCHuh7m; mcherry- (m-): Huh7R, NCCHuh7, CCHuh7R. **J** Quantitative statiststics of ratio (m+/m-) in (**F**). Three independent experiments were conducted, and the values are represented by means ± SEM using *t*-test (**C**, **G**) or two-way ANOVA with Tukey’s multiple comparisons test (**E**, **J**). *p < 0.05, ns non-statistically significant. See also Figs. S1 and S2.

To characterize the biological behavior conferred by lenvatinib resistance of HCC cells, we established successfully the lenvatinib-resistant HCC cell strain (Huh7R; IC50 = 40.91 μM) with the resistance characteristic of high expression in receptor protein tyrosine kinase (RPTKs) by culturing Huh7 cells (IC50 = 3.45 μM) with progressively increased doses of lenvatinib (3–30μm) for 6 months following the published studies [5, 17] (Supplemental Fig. S1A–D; Video 1–4); meanwhile, a stable mCherry-tagged Huh7 cell strain (Huh7m) displayed no significant difference from Huh7 in terms of cell migration, proliferation, apoptosis, and intracellular reactive oxygen species (ROS) level was developed and incorporated into the co-culture system (Supplemental Fig. S1E–M). We chose Huh7 cell line mainly based on the findings that Huh7 shows a significantly active Activity Area (AA) in common with other HCC cell lines as well as a strong correlation under lenvatinib treatment, through analyzing the AA of 90 anti-cancer drugs on 81 LIMORE HCC cell models [18] (Supplemental Fig. S2A, B), which suggests Huh7 harbors the representativity and universality of studying lenvatinib resistant HCC. In addition, we enrolled lenvatinib-resistant PLC-PRF-5 cell (PLC-PRF-5R; IC50 = 55.2 μM) for further congeners verification (Supplemental Fig. S1N). Thereafter, HCC cell competition models in vitro by coculturing Huh7R with Huh7m, and PLC-PRF-5R with PLC-PRF-5m at a 1:1 ratio (defined cell competition group [CC group], in which Huh7R/PLC-PRF-5R and Huh7m/PLC-PRF-5m, were respectively termed as CCHuh7R/CCPLC-PRF-5R and CCHuh7m/CCPLC-PRF-5m); meanwhile, a non-competition group [NCC group] was set up by coculturing Huh7 with Huh7m, and PLC-PRF-5 with PLC-PRF-5m at a 1:1 ratio, in which Huh7/PLC-PRF-5 and Huh7m/PLC-PRF-5m were respectively termed as NCCHuh7/NCCPLC-PRF-5 and NCCHuh7m/NCCPLC-PRF-5m. Cell competition phenotype was evaluated based on the combination of flow cytometric analysis and visualization with high content imaging analysis (Fig. 1A). In CC group, CCHuh7m suffered growth inhibition and cell death, while CCHuh7R obtained concomitantly increased growth (Fig. 1B; Video 5). To ascertain whether such a biological behavior occurs in vivo, we analyzed the percent change of mCherry positive-cells from NCC and CC groups through constructing subcutaneously implanted tumor models in nude mice. Similarly, the outcompeting of lenvatinib-resistant cells (Huh7R/PLC-PRF-5R) against lenvatinib-sensitive cells (Huh7m/PLC-PRF-5m) was observed in xenograft tumors from CC group but not NCC group, which further indicated the potential existence of cell competition between lenvatinib-resistant HCC cells and sensitive HCC cells (Fig. 1C; Supplemental Fig. S1O). To prove that the elimination of CCHuh7m indeed results from cell competition, we compared firstly the growth rates of a variety of cells in CC group (CCHuh7R, CCHuh7m), NCC group (NCCHuh7, NCCHuh7m) and alone culture groups (Huh7, Huh7m, Huh7R). Although there existed a different growth rate baseline that Huh7R cells grew faster than Huh7m or Huh7 cells [19, 20], as expected, CCHuh7R cells grew significantly faster than Huh7R, while CCHuh7m grew slower than Huh7m or NCCHuh7m cells, even experienced cell death and there was no difference among NCCHuh7m, NCCHuh7, Huh7m and Huh7 cells (Fig. 1D, E; Video 6–10). Similarly, cell competition-mediated growth advantage of CCHuh7R was also observed in the liver orthotopic model (Fig. 1F–H). Moreover, the population ratios (m + /m-) displayed a gradually descending trend in NCC group, mixed group (the mixture of Huh7m/PLC-PRF-5m with Huh7R/PLC-PRF-5R cultured alone as a control) and CC group at indicated 24 h/48 h, or specifically in CC group over a time course of coculture (Fig. 1I, J; Supplemental Fig. S1P, Q); the result confirmed the cell competition phenomenon observed in the imaging analysis above. Together, these results suggest that there exists cell competition between lenvatinib-resistant HCC cells and sensitive HCC cells under coculture condition and importantly the cell competition behavior further endows HCC lenvatinib-resistant cells with a growth advantage at the price of lenvatinib-sensitive cells.

### Lenvatinib-resistant cells present transcriptionally upregulated glycolytic metabolism in cell competition condition

**Fig. 2:**
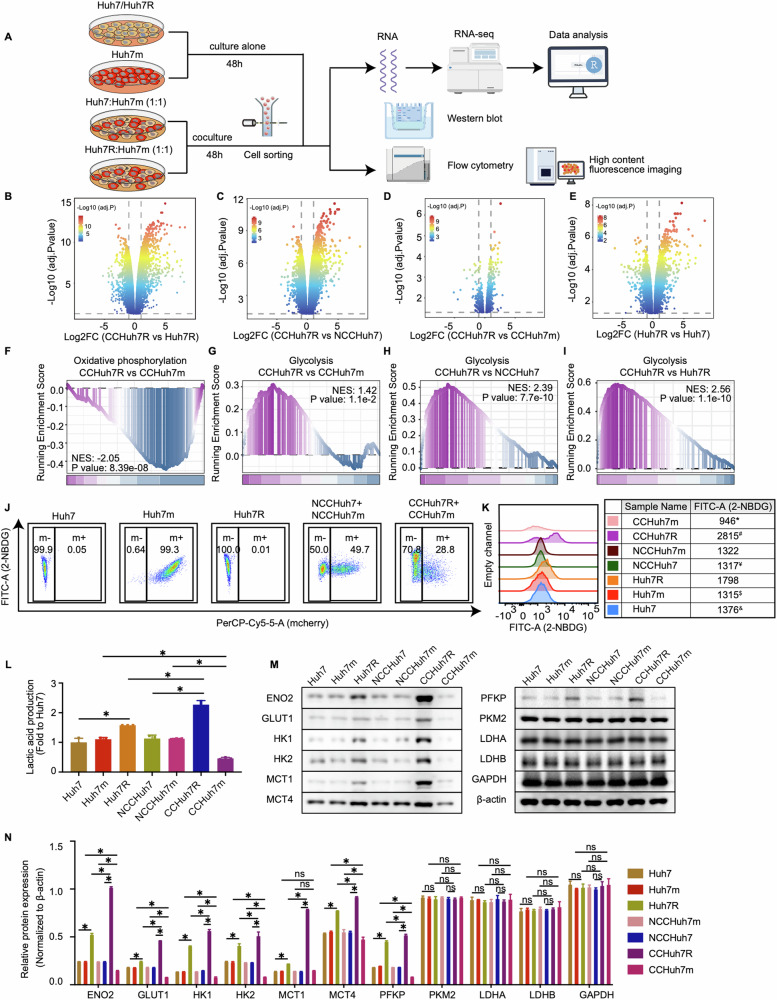
Lenvatinib-resistant cells display upregulated glycolytic metabolism in cell competition at transcriptomic level. **A** Schematic workflow of our experimental strategy by Figdraw. Huh7, Huh7m and Huh7R cultured alone; Huh7 or Huh7R cocultured with Huh7m at 1:1 ratio respectively. Cells were cultured for 48 h after cell attachment and then coculture groups were separated using flow cytometry sorting for RNA-seq, Western blot and other purposes. **B** Volcano plot showing differentially expressed genes in CCHuh7R vs CCHuh7R group. **C** As in (**B**) but in CCHuh7R vs NCCHuh7 group. **D** As in (**B**) but in CCHuh7R vs CCHuh7m group. **E** As in (**B**) but in Huh7R vs Huh7 group. **F** GSEA analysis of oxidative phosphorylation enrichment in CCHuh7R vs CCHuh7m group. **G** GSEA analysis of glycolysis enrichment in CCHuh7R vs CCHuh7m group. **H** As in (**G**) but in CCHuh7R vs NCCHuh7 group. **I** As in (**G**) but in CCHuh7R vs Huh7R group. **J** Flow cytometry analysis of 2-NBDG-FITC-A and cell proportion at 48 h after cell attachment in Alone group, NCC group and CC group. **K** Mountain map of 2-NBDG-FITC-A in (**J**). * CCHuh7m vs NCCHuh7m (*p* < 0.05); $ CCHuh7m vs Huh7m (*p* < 0.05); ¥ CCHuh7R vs NCCHuh7 (*p* < 0.05); # CCHuh7R vs Huh7R (*p* < 0.05); & Huh7R vs Huh7 (*p* < 0.05). **L** Cellular lactic acid production levels at 48 h after cell attachment in Alone group, NCC group and CC group by flow cytometry cell sorting. **M** Protein levels of ENO2, GLUT1, HK1, HK2, MCT1, MCT4, PFKP, PKM2, LDHA, LDHB and GAPDH at 48 h after cell attachment in Alone group, NCC group and CC group detected by western blot. **N** Quantitative statistics of protein levels in (**M**). Three independent experiments were conducted, and the values are represented by means ± SEM using two-way ANOVA with Tukey’s multiple comparisons test. *p < 0.05, #*p* < 0.05, $*p* < 0.05, ¥*p* < 0.05, &*p* < 0.05, ns non-statistically significant. See also Fig. S3.

To uncover the biological mechanism of underlying cell competition between lenvatinib-resistant HCC cells and sensitive HCC cells, we separated the cocultured cells including CCHuh7R/CCHuh7m and NCCHuh7/NCCHuh7m by performing a flow cytometry cell sorting followed by RNA sequencing (RNA-seq) and bioinformatics analysis; meanwhile alone cultured Huh7, Huh7m and Huh7R were subjected to the same processing (Fig. 2A). According to GSEA analysis based on the results of differential expression analysis (Fig. 2B–E), oxidative phosphorylation was significantly down-regulated in CCHuh7R compared to CCHuh7m cells (Fig. 2F). Of note, the decreased enrichment of mitochondria mass or oxidative phosphorylation related activities (mitochondrial respiratory chain complex assembly, mitochondrial gene expression, mitochondrial transmembrane transport, mitochondrial translation, mitochondrial RNA metabolic process, oxidative phosphorylation) was also found in CCHuh7R vs NCCHuh7/CCHuh7m and Huh7R vs Huh7 (Supplemental Fig. S3A–F). Considering the change feature of growth rate as described above (see Fig. 1D, E; Video 6–10) and the down-regulated oxidative phosphorylation in CCHuh7R cells under cell competition condition, we inferred that there may exist a significantly altered energy metabolism activity contributing to cell competition phenotype of resistant HCC cells. Consistent with our hypothesis, further GSEA analysis showed that glycolysis was significantly enriched and up-regulated in CCHuh7R cells compared to CCHuh7m cells, NCCHuh7 or Huh7R cells (Fig. 2G–I); while no significant metabolism-related pathways were enriched in NCCHuh7 cells vs NCCHuh7m or Huh7m or Huh7 cells. The data above indicated that energy metabolism reprogramming may be involved in the cell competition between lenvatinib-resistant cells and sensitive cells in HCC.

To further confirm the findings from RNA-seq analysis, we examined the levels of cellular 2-NBDG, the indigestible glucose analog as a marker for glycolysis [21, 22], in cells from a myriad of models in vitro. Notably, both CCHuh7R and CCPLC-PRF-5R displayed higher 2-NBDG level than NCCHuh7/NCCPLC-PRF-5 or Huh7R/PLC-PRF-5R cells; in contrast, CCHuh7m cells presented significantly decreased 2-NBDG level compared to NCCHuh7m or Huh7m cells (Fig. 2J, K; Supplemental Fig. S3G, H). Additionally, the end-product lactic acid of the glycolysis process was detected to determine holistic glycolytic activity. Consistent with the 2-NBDG results, the level of intracellular lactic acid was significantly higher in CCHuh7R than in NCCHuh7 or Huh7R cells but relatively lower in CCHuh7m than in NCCHuh7m or Huh7m cells (Fig. 2L). Considering the fact that metabolic substrates and glycolysis activity were increased, we further detected some glycolysis-related key metabolic proteins including ENO2, GLUT1, HK1, HK2, MCT1, MCT4, PFKP and found that these proteins were highly expressed in CCHuh7R compared to NCCHuh7 or Huh7R cells; in contrast, CCHuh7m displayed lower expressions of glycolysis-related metabolic proteins compared to NCCHuh7m or Huh7m cells (Fig. 2M, N). These results confirmed the hints from our bioinformatic analysis. Collectively, it indicates that enhanced glycolysis of lenvatinib-resistant cells may contribute to its outcompeting against sensitive cells in HCC.

As is well-known, the Warburg effect is recognized as the hallmark of tumor [21], and tumor cells as a dominant population obtain adequate nutrients for advantage proliferation and better survival in the stress microenvironment via energy metabolism reprogramming [22, 23]. Thus, to reveal the association between energy metabolism and cell competition in HCC, we first examined the mitochondrial membrane potential by using Rhodamine 123, a positively charged green fluorescent dye that aggregated in active mitochondria and determined by a membrane potential gradient through their inner membranes. The result showed that CCHuh7R cells exhibited significantly lower mitochondrial membrane potential than NCCHuh7 or Huh7R cells; moreover, the mitochondrial membrane potential was also decreased in CCHuh7m compared to those in Huh7m/NCCHuh7m cells, potentially underlying the incompetent status of CCHuh7m as a loser, though its oxidative phosphorylation levels were higher than CCHuh7R (Supplemental Fig. S3I, J). Furthermore, Mito-Tracker Green, a mitochondrial morphology indicator, was used and it was found that energy metabolism shift was significantly observed in the cell competition model and decreased mitochondrial mass of CCHuh7R cells compared to NCCHuh7 or Huh7R cells (Supplemental Fig. S3K, M, N), which was further confirmed by the results of transmission electron microscope (TEM) (Supplemental Fig. S3L). Of note, the mitochondria of CCHuh7R displayed morphologically grainy that was exacerbated in a time-dependent manner (Supplemental Fig. S3O), which was consistent with the mitochondria morphology of mitophagy process [24]. Further, lower expression levels of IDH3A, COX7A, SDHA, UQCRC2, OGDH and CS proteins, which are rate-limiting enzymes in oxidative phosphorylation, were found in CCHuh7R cells compared to NCCHuh7 or Huh7R cells; while CCHuh7m exhibited significantly higher expression levels of these proteins than NCCHuh7m or Huh7m cells (Supplemental Fig. S3P, Q). Altogether, all these findings suggested that energy metabolism reprogramming may serve as a trigger of cell competition, in which mitochondrial oxidative phosphorylation attenuates while glycolysis enhances in lenvatinib-resistant cells, thereby contributing to drug resistance in HCC.

### Increased glycolytic flux supports the winner status of lenvatinib-resistant HCC cells

**Fig. 3:**
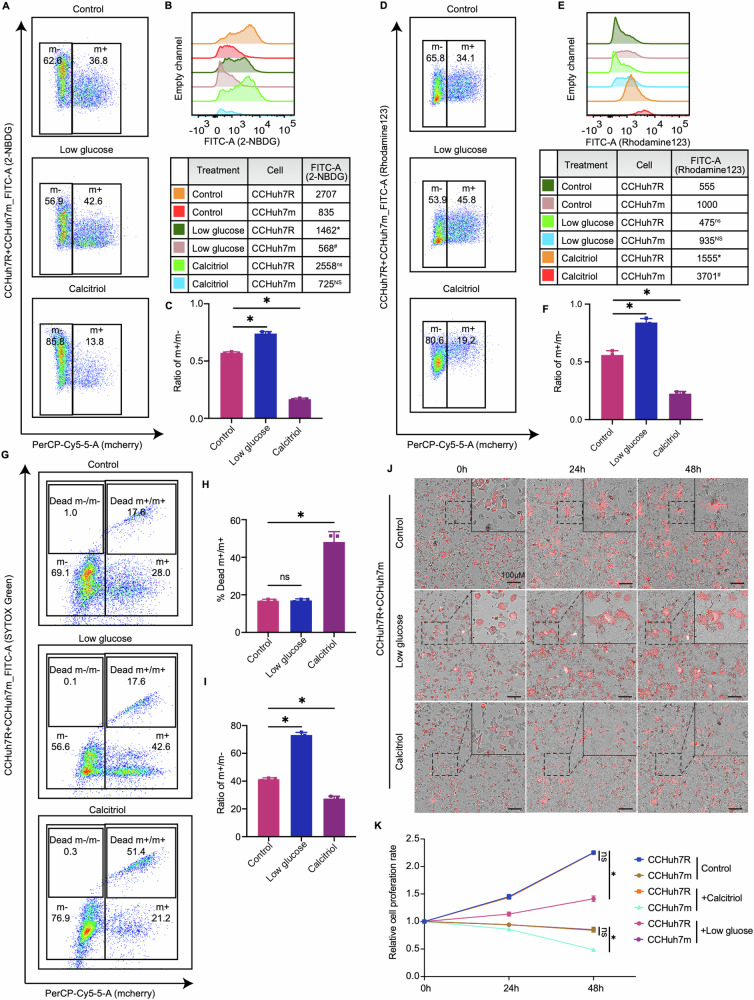
Enhanced glycolysis supports the winner status of lenvatinib-resistant cells. **A** Flow cytometry analysis of 2-NBDG-FITC-A and cell proportion at 48 h after cell attachment in CC group with low glucose or Calcitriol treatment. **B** Quantitative statistics of 2-NBDG-FITC-A intensity in (**A**). * CCHuh7R (Low glucose) vs CCHuh7R (Control) (*p* < 0.05); # CCHuh7m (Low glucose) vs CCHuh7m (Control) (*p* < 0.05); ns CCHuh7R (Calcitriol) vs CCHuh7R (Control) (*p* > 0.05); NS CCHuh7m (Calcitriol) vs CCHuh7m (Control) (*p* > 0.05). **C** Quantitative statistics of ratio of m + /m- in (**A**). **D** Flow cytometry analysis of mitochondrial membrane potential (Rhodamine123-FITC-A) and cell proportion at 48 h after cell attachment in CC group with low glucose or Calcitriol treatment. **E** Quantitative statistics of 2-NBDG-FITC-A in (**D**). ns CCHuh7R (Low glucose) vs CCHuh7R (Control) (*p* > 0.05); NS CCHuh7m (Low glucose) vs CCHuh7m (Control) (*p* > 0.05); * CCHuh7R (Calcitriol) vs CCHuh7R (Control) (*p* < 0.05); # CCHuh7m (Calcitriol) vs CCHuh7m (Control) (*p* < 0.05). **F** Quantitative statistics of ratio of m + /m- in (**D**). **G** Flow cytometry analysis of cell death (SYTOX Green-FITC-A) and cell proportion at 48 h after cell attachment in CC group with low glucose or Calcitriol treatment. **H** Quantitative statistics of Dead m + /m+ ratio in (**G**). **I** Quantitative statistics of m + /m+ ratio in (**G**). **J** High content imaging of cell growth at 0, 24 h, 48 h after cell attachment in CC group with low glucose or Calcitriol treatment using 10X objective lens. **K** Quantitative statistics of cell number in (**J**) (Details see Video 11–13). Three independent experiments were conducted, and the values are represented by means ± SEM using two-way ANOVA with Tukey’s multiple comparisons test (**B**, **E** and **K**) or Ordinary one-way ANOVA with Sidak’s multiple comparisons test (**C**, **F**, **H** and **I**). *p < 0.05, #*p* < 0.05, ns/NS non-statistically significant.

As indicated by our transcriptomics analysis and experiments above, energy metabolism reprogramming contributes to cell competition between CCHuh7R and CCHuh7m cells. Therefore, we further investigated the effect of low glucose on cell competition behavior between CCHuh7R and CCHuh7m cells. Compared with the control group, low glucose led to the decreased 2-NBDG uptake of CCHuh7R cells but no change in mitochondrial membrane potential, as observed by flow cytometry; notably, unaltered cell population dominance of CCHuh7R (m + /m- < 1) compared with the untreated CCHuh7R (m + /m- < 1) indicated that cell competition phenomenon was weakened (m + /m-: Low glucose group > Control group; *p* < 0.05) but not abolished (m + /m- < 1) in low glucose condition. Subsequently, to assess the effect of oxidative phosphorylation on cell competition, we added Calcitriol (1,25-Dihydroxyvitamin D3), an oxidative phosphorylation agonist reagent [25, 26], into the cell competition model and found that 2-NBDG uptake in CCHuh7R cells was not significantly changed in contrast to the control one. Interestingly, the treatment of Calcitriol not only significantly increased mitochondrial membrane potential of CCHuh7R but also aggravated its dominance status compared to the control group (m + /m- ratio; Calcitriol group<Control group: *p* < 0.05) (Fig. 3A–F), indicating that cell competition behavior was further enhanced. These results preliminarily suggest that both the energy restriction such as low glucose and the regulation of mitochondria oxidative phosphorylation metabolic levels may have a considerable but opposite impact on cell competition.

To further identify the detailed effects of energy metabolism reprogramming on cell competition in HCC, we performed a SYTOX Green assay to observe the change of CCHuh7m/CCHuh7R ratio with specific treatments in a cell competition scenario. Noticeably, low glucose treatment had no significant effect on the death ratios of CCHuh7m (dead CCHuh7m vs CCHuh7m) cells and CCHuh7R (Dead CCHuh7R vs CCHuh7R) cells, but the ratio of CCHuh7m /CCHuh7R remarkably increased. However, upon Calcitriol treatment, more than 2-fold was observed in the death ratio of CCHuh7m cells, accompanied by unaltered death ratio of CCHuh7R cells but reduced the ratio of CCHuh7m/CCHuh7R cells (Fig. 3G–I). These results imply that the variation of cell competition between CCHuh7R and CCHuh7m cells most likely depends on their distinct metabolic features. Thus, we then examined and compared the growth rates of CCHuh7R and CCHuh7m cells under the above additions in a setting of cell competition. As expected, low glucose treatment mainly attenuated the growth of CCHuh7R cells but did not strongly suppress the growth of CCHuh7m cells. Nevertheless, Calcitriol profoundly reduced the viability of CCHuh7m cells but had no notable effect on the growth of CCHuh7R cells (Fig. 3J, K; Video 11–13). These results showed energy restriction resulted in the increase of the CCHuh7m/CCHuh7R ratio by inhibiting CCHuh7R growth, whereas the activated oxidative phosphorylation levels led to the diminishment of the CCHuh7m/CCHuh7R ratio by increasing the death of CCHuh7m cells.

Collectively, these in vitro studies above prove that enhanced glycolysis, but not oxidative phosphorylation, is the principal competitive strategy by maintaining the winner status of lenvatinib-resistant HCC cells.

### BNIP3-mediated mitophagy is required for enhanced glycolysis of lenvatinib-resistant cells (winner)

**Fig. 4:**
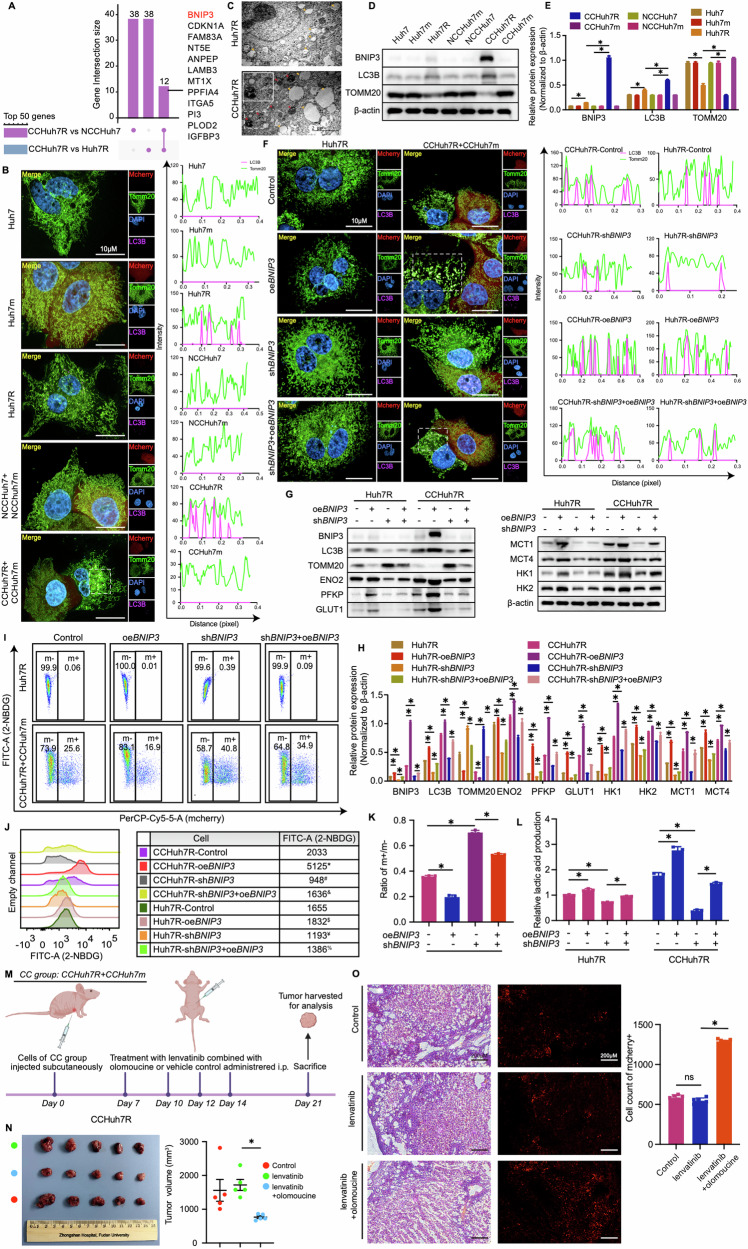
Enhanced glycolytic flux of lenvatinib-resistant cells (winner) requires BNIP3-mediated mitophagy. **A** UpSet plot showing Top 50 DEGs’ overlapping genes in CCHuh7R vs Huh7R group and CCHuh7R vs NCCHuh7 group. **B** (Left) High content immunofluorescence imaging of colocalization of autophagosomes (Cy5.5-LC3: purple) and mitochondria (TOMM20: green) at 48 h after cell attachment in Alone group, NCC group and CC group using 63X water immersion objective lens. (Right) Quantification of colocalization between LC3 (purple peak) and TOMM20 (green peak) in the above groups. Purple/green peak height represents fluorescence intensity of LC3B/TOMM20; overlapping peaks indicate colocalization numbers between LC3B and Tomm20. **C** Transmission electron microscope imaging of Huh7R and CCHuh7R, the arrow (orange) indicates mitochondria of cell, the arrow (red) represents mitophagy activity in cell. The enlargement picture of the dashed boxes in CCHuh7R shows the details of specific mitophagy activity. **D** Protein levels of LC3B, TOMM20 and BNIP3 at 48 h after cell attachment in Alone group, NCC group and CC group detected by western blot. **E** Quantitative statistics of protein levels in (**C**). **F** (Left) High content immunofluorescence imaging of colocalization of autophagosomes (Cy5.5-LC3: purple) and mitochondria (TOMM20: green) at 48 h after cell attachment in Huh7R (Alone group) /CCHuh7R (CC group) transfected with shRNA against *BNIP3* (sh*BNIP3*), *BNIP3* overexpression plasmid (oe*BNIP3*) or oe*BNIP3*+sh*BNIP3* using 63X water immersion objective lens. (Right) Quantification of colocalization between LC3 (purple peak) and TOMM20 (green peak) in the above groups. Purple/green peak height represents fluorescence intensity of LC3B/TOMM20; overlapping peaks indicate colocalization numbers between LC3B and Tomm20. **G** Protein levels of LC3B, TOMM20, BNIP3, ENO2, GLUT1, HK1, HK2, MCT1, MCT4 and PFKP at 48 h after cell attachment in Huh7R (Alone group) /CCHuh7R (CC group) transfected with sh*BNIP3*, oe*BNIP3* or sh*BNIP3*+oe*BNIP3* detected by western blot. **H** Quantitative statistics of protein levels in (**H**). **I** Flow cytometry analysis of 2-NBDG-FITC-A and cell proportion at 48 h after cell attachment in Huh7R (Alone group) /CCHuh7R (CC group) transfected with sh*BNIP3*, oe*BNIP3* or sh*BNIP3*+oe*BNIP3*. **J** Mountain map of 2-NBDG-FITC-A in (**J**). * CCHuh7R-oe*BNIP3* vs CCHuh7R-Control (*p* < 0.05); # CCHuh7R-sh*BNIP3* vs CCHuh7R-Control (*p* < 0.05); & CCHuh7R-sh*BNIP3+*oe*BNIP3* vs CCHuh7R-sh*BNIP3* (*p* < 0.05); $ Huh7R-oe*BNIP3* vs Huh7R-Control (*p* < 0.05); ¥ Huh7R-sh*BNIP3* vs Huh7R-Control (*p* < 0.05); % Huh7R-sh*BNIP3+*oe*BNIP3* vs Huh7R-sh*BNIP3* (*p* < 0.05). **K** Quantitative statistics of ratio m + /m- in (**J**). **L** Cellular lactic acid production levels at 48 h after cell attachment in Huh7R (Alone group) /CCHuh7R (CC group) transfected with sh*BNIP3*, oe*BNIP3* or sh*BNIP3*+oe*BNIP3* by flow cytometry cell sorting. (**M**-**N**) Schematic workflow (**M**), tumor photos and statistic analysis of tumor volume (**N**) in our HCC subcutaneous model under lenvatinib treatment with or without olomouine. *N* = 5. **O** (Left) Representative H&E stain and fluorescence imaging of cells in CC group-mice with or without lenvatinib or lenvatinib+olomouine. (Right) Mcherry positive cells in corrresponding groups were counted with Image (**J**). *N* = 5. Three independent experiments were conducted, and the values are represented by means ± SEM using two-way ANOVA with multiple comparisons test (**E**, **H**, **L**, **J** and **M**) or Ordinary one-way ANOVA with Sidak’s multiple comparisons test (**K**, **N**, **O**). *p < 0.05, #*p* < 0.05, $*p* < 0.05, ¥*p* < 0.05, &*p* < 0.05, %*p* < 0.05, ns non-statistically significant. See also Figs. S4 and S5.

To further clarify the molecular mechanism by which enhanced glycolysis activity of CCHuh7R contributed to its cell competition advantage in our model, we analyzed the differentially expressed genes (DEGs) from CCHuh7R vs Huh7R group and CCHuh7R vs NCCHuh7 group. UpSet plot analysis identified twelve overlapping genes in Top 50 DEGs of each group which were ranked by FDR (Fig. 4A), demonstrating that these genes’ expressions were remarkably changed in cell competition scenario. Interestingly, BNIP3, a marker of mitophagy occurrence and its participation in improving glycolysis activities including glucose uptake and lactic acid production [27–29], exhibited the most significant up-regulation (Fig. 4A). Considering the findings on attenuated mitochondrial oxidative phosphorylation in CCHuh7R cells validated by GSEA analysis and cellular functional experiments, we speculated that enhanced glycolytic flux of lenvatinib-resistant HCC cells might be associated with BNIP3-mediated mitochondria dysfunctions. To validate this hypothesis, a series of bioinformatic analyses and relevant experiments were performed. As expected, CCHuh7R cells showed significant down-regulated mitochondrial mass/activities by GSVA analysis and differential pathway analysis based on a customized gene set of GO and, surprisingly, two mitophagy-related activities (positive regulation of mitophagy in response to mitochondrial depolarization, regulation of autophagy of mitochondrion in response to mitochondrial depolarization) were notably enriched in these activities (Supplemental Fig. S4A, B). Furthermore, the identification of colocalization between Cy5.5-LC3B-positive autophagosome and TOMM20-tagged mitochondria detected by immunofluorescence showed that there was colocalization in CCHuh7R and Huh7R cells and it occurred significantly more in CCHuh7R than in Huh7R cells (Fig. 4B). Importantly, as a winner in cell competition, CCHuh7R and CCPLC-PRF-5R exhibited more outstanding mitophagy activities for eliminating aberrant mitochondria in TEM when compared with Huh7R or PLC-PRF-5R (Fig. 4C; Supplemental Fig. S4C) which was consistent with reports in sorafenib resistant HCC cells [30, 31]; moreover, higher expression of mitophagy-related protein levels (LC3B, BNIP3) and lower expression of TOMM20 representing mitochondria mass were further observed in cells (Fig. 4D, E; Supplemental Fig. S4D, E). Based on the above findings, we specifically focused on the mitophagy pathway and found that BNIP3 with the minimum FDR displayed remarkable expression difference in two comparative groups as shown by heatmap (Supplemental Fig. S4F, G). These data again substantiated our hypothesis. Although we have identified the occurrence of mitochondrial aberration and mitophagy of lenvatinib-resistant HCC cells in cell competition scenario, the correlation between BNIP3-mediated mitophagy and glycolysis was still not completely clear. Therefore, clinical HCC specimens in TCGA and ICGC databases (TCGA-LIHC cohort and ICGC-LIHC cohort) were firstly analyzed and categorized into BNIP3 high-expression and low-expression groups according to the median value of BNIP3 expression. GSEA analyses from both cohorts showed that glycolysis pathway was remarkably enriched and up-regulated in the BNIP3 high-expression group and had a positive correlation with BNIP3 expression based on ssGSEA analysis (Supplemental Fig. S5A-D).

To further prove the findings above from bioinformatic analyses, we constructed a variety of genetically modified Huh7R/PLC-PRF-5R cells with *BNIP3* overexpression (oe*BNIP3*) or *BNIP3* knockdown (sh*BNIP3*) followed by the coculture of Huh7m/ PLC-PRF-5m (Huh7R-sh*BNIP3*, Huh7R-oe*BNIP3*, CCHuh7R-sh*BNIP3*, CCHuh7R-oe*BNIP3*; PLC-PRF-5R-sh*BNIP3*, PLC-PRF-5R-oe*BNIP3*, CCPLC-PRF-5R-sh*BNIP3*, CCPLC-PRF-5R-oe*BNIP3*). Considering the rigor and logicality of validation, metabolic indicators of modified cells were also investigated under another experimental manipulation where the expression of BNIP3 protein was induced in a mosaic form by transient expression (Huh7R-sh*BNIP3*+oe*BNIP3*, CCHuh7R-sh*BNIP3*+oe*BNIP3*; PLC-PRF-5R-sh*BNIP3*+oe*BNIP3*, CCPLC-PRF-5R-sh*BNIP3*+oe*BNIP3*). We found that the knockdown of *BNIP3* significantly inhibited the colocalization of Cy5.5-LC3B and TOMM20 in CCHuh7R/CCPLC-PRF-5R cells (Fig. 4F; Supplemental Fig. S5E). Furthermore, CCHuh7R-sh*BNIP3* relative to Huh7R-sh*BNIP3* expressed higher TOMM20, which could be rescued by the transient transfection of *BNIP3*. Conversely, *BNIP3* overexpression achieved opposite effects (Fig. 4G, H). These results indicated the importance of BNIP3 which induces the occurrence of mitophagy for eliminating abnormal mitochondria in co-cultured resistant cells, although there existed a moderate change in cultured alone-resistant cells in line with what has been reported previously [32]. Noteworthily, *BNIP3* knockdown decreased the expression levels of glycolysis-related proteins (ENO2, GLUT1, HK1, HK2, MCT1, MCT4, PFKP) as well as 2-NBDG and lactic acid production in these cells; however, this manipulation displayed positive but limited alteration of the competitive advantage of CCHuh7R/CCPLC-PRF-5R cells (Fig. 4G–L; Supplemental Fig. S5F–I). In addition, we evaluated the effect of BNIP3 on cell competition through treating the cell competition xenograft models in vivo with BNIP3 inhibitor olomoucine (Fig. 4M) and found that the specific inhibition of BNIP3 effectively suppressed tumor growth (Fig. 4N, O; Supplemental Fig. S5J). Altogether, we concluded that lenvatinib-resistant HCC cells hold the increased glycolytic flux through BNIP3-mediated mitophagy, thereby maintaining its winner status.

### BNIP3-regulated ENO2 orchestrates the effects of mitophagy on glycolysis of lenvatinib-resistant HCC cells

**Fig. 5:**
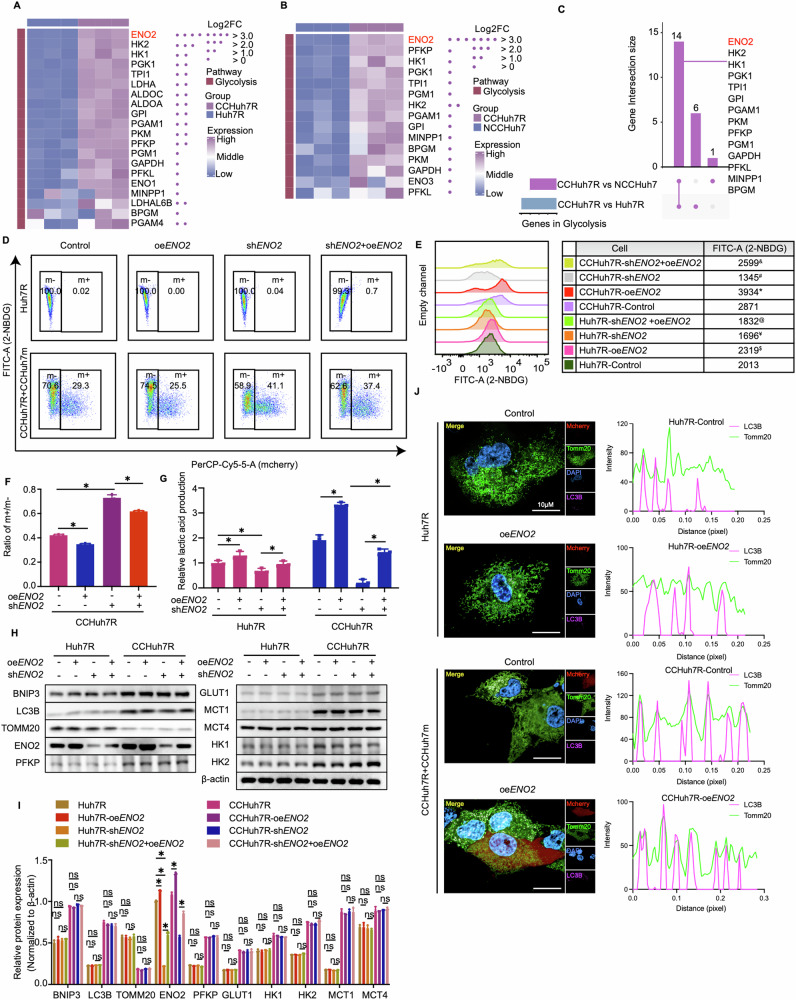
The effect of glycolysis of winner is ENO2 dependent. **A** Heatmap exhibiting expression levels of glycolysis-related genes between CCHuh7R and Huh7R. **B** As in (**A**) but between CCHuh7R and NCCHuh7. **C** UpSet plot displaying overlapping glycolysis-related genes based on (**A**) and (**B**). **D** Flow cytometry analysis of 2-NBDG-FITC-A and cell proportion at 48 h after cell attachment in Huh7R (Alone group) /CCHuh7R (CC group) transfected with shRNA against *ENO2* (sh*ENO2*), *ENO2* overexpression plasmid (oe*ENO2*) or oe*ENO2*+sh*ENO2*. **E** Mountain map of 2-NBDG-FITC-A in (**D**). * CCHuh7R-oe*ENO2* vs CCHuh7R-Control (*p* < 0.05); # CCHuh7R-sh*ENO2* vs CCHuh7R-Control (*p* < 0.05); & CCHuh7R-sh*ENO2+*oe*ENO2* vs CCHuh7R-sh*ENO2* (*p* < 0.05); $ Huh7R-oe*ENO2* vs Huh7R-Control (*p* < 0.05); ¥ Huh7R-sh*ENO2* vs Huh7R-Control (*p* < 0.05); % Huh7R-sh*ENO2+*oe*ENO2* vs Huh7R-sh*ENO2* (*p* < 0.05). **F** Quantitative statistics of ratio m + /m- in (**D**). **G** Cellular lactic acid production levels at 48 h after cell attachment in Huh7R (Alone group) /CCHuh7R (CC group) transfected with sh*ENO2*, oe*ENO2* or sh*ENO2*+oe*ENO2* by flow cytometry cell sorting. **H** Protein levels of LC3B, TOMM20, BNIP3, ENO2, GLUT1, HK1, HK2, MCT1, MCT4 and PFKP at 48 h after cell attachment in Huh7R (Alone group) /CCHuh7R (CC group) transfected with sh*ENO2*, oe*ENO2* or sh*ENO2*+oe*ENO2* detected by western blot. **I** Quantitative statistics of protein levels in (**H**). **J** (Left) High content immunofluorescence imaging of colocalization of autophagosomes (Cy5.5-LC3: purple) and mitochondria (TOMM20: green) at 48 h after cell attachment in Huh7R (Alone group) /CCHuh7R (CC group) transfected with sh*ENO2*, oe*ENO2* or sh*ENO2*+oe*ENO2* using 63X water immersion objective lens. (Right) Quantification of colocalization between LC3 (purple peak) and TOMM20 (green peak) in the above groups. Purple/green peak height represents fluorescence intensity of LC3B/TOMM20; overlapping peaks indicate colocalization numbers between LC3B and Tomm20. Three independent experiments were conducted, and the values are represented by means ± SEM using a two-way ANOVA with Tukey’s multiple comparisons test (**E**, **G** and **I**) or Ordinary one-way ANOVA with Sidak’s multiple comparisons test (**F**). *p < 0.05, #*p* < 0.05, $*p* < 0.05, ¥*p* < 0.05, &*p* < 0.05, %*p* < 0.05, ns = non-statistically significant. See also Fig. S5.

Next, we sought to uncover the mechanism how mitophagy regulates glycolysis or whether there exists a major glycolysis target molecular regulated by mitophagy. In the previous western blot results, we noticed that ENO2, a key enzyme of glycolysis process, showed a distinct variation in malignant phenotype-related glycolysis metabolism of CCHuh7R cells (see Fig. 2M, N; Fig. 4G, H), which is consistent with reports in cancers [33–35]. Of note, a specific glycolysis pathway analysis also showed that ENO2 with the minimum FDR displayed the biggest expression difference in CCHuh7R vs Huh7R group and CCHuh7R vs NCCHuh7 group depicted by heatmap and UpSet plot (Fig. 5A–C). The above results potentially implied that ENO2 may be a critical effector in the competitive cascade response of CCHuh7R cells to cell competition scenario. Therefore, we accordingly tested whether ENO2 exerts a specific effect on glycolysis activity and competitive behavior of lenvatinib-resistant HCC cells. Strikingly, we found that *ENO2* deficiency (sh*ENO2*) specifically contributed to dramatic decrease in 2-NBDG (Fig. 5D, E; Supplemental Fig. S6A, B) and lactic acid production of CCHuh7R/CCPLC-PRF-5R (CCHuh7R-sh*ENO2*; CCPLC-PRF-5R-sh*ENO2*) cells, which could be abrogated by the transient supplement of ENO2 (CCHuh7R-sh*ENO2*+oe*ENO2*; CCPLC-PRF-5R-sh*ENO2*+oe*ENO2*); corresponding changes were observed in the administration of ENO2 overexpression (CCHuh7R-oe*ENO2*; CCPLC-PRF-5R-oe*ENO2*) (Fig. 5G; Supplemental Fig. S6D). Nevertheless, both cells exhibited no remarkable change in glycolysis and mitophagy-related proteins expressions (Fig. 5H, I) and the colocalization between Cy5.5-LC3B and TOMM20 (Fig. 5J), no matter which genetic modification was performed (sh*ENO2*, oe*ENO2*). These results represented ENO2 functions as an unique role in enhanced glycolysis activity of CCHuh7R/CCPLC-PRF-5R cells, as a downstream of BNIP3. Of note, the ratio of m + /m-(CCHuh7m/CCHuh7R; CCPLC-PRF-5m/CCPLC-PRF-5R) obtained a marked but limited increase even in the case of the reduction of ENO2-represented glycolysis levels of CCHuh7R/CCPLC-PRF-5R cells (Fig. 5F; Supplemental Fig. S6C), potentially manifesting that the variation of ENO2 expression is still not efficient enough to eliminate cell competition phenomenon. To elucidate further the detailed relationship between mitophagy, glycolysis and ENO2, we overexpressed BNIP3 in *ENO2*-deficient Huh7R/PLC-PRF-5R followed by the coculture with Huh7m/PLC-PRF-5m (Huh7R-sh*ENO2*+oe*BNIP3*, CCHuh7R-sh*ENO2*+oe*BNIP3*; PLC-PRF-5R-sh*ENO2*+oe*BNIP3*, CCPLC-PRF-5R-sh*ENO2*+oe*BNIP3*). As expected, *BNIP3*-temporary overexpression rescued the detected effects of CCHuh7R-sh*ENO2*/CCPLC-PRF-5R-sh*ENO2* cells, which was reasonably inferior to CCHuh7R-oe*BNIP3*/CCPLC-PRF-5R-oe*BNIP3* cells (Supplemental Fig. S6E–L). These results suggested that there existed BNIP3-ENO2 regulatory axis which featured in enhanced glycolysis of CCHuh7R/ CCPLC-PRF-5R cells in cell competition scenario.

### AMPK functions as a signaling bridge in the crosstalk between BNIP3 and ENO2 in lenvatinib-resistant HCC cells

**Fig. 6:**
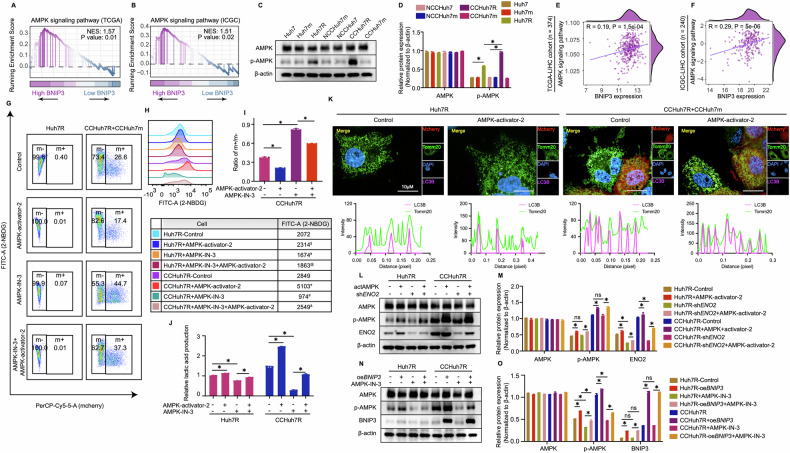
AMPK functions as a signaling bridge in BNIP3-ENO2 crosstalk in lenvatinib-resistant cells. **A** GSEA analysis of AMPK signaling pathway enrichment in BNIP3 high expression vs BNIP3 low expression group of TCGA-LIHC cohort. **B** As in (**A**) but in ICGC-LIHC cohort. **C** Protein levels of AMPK and p-AMPK at 48 h after cell attachment in Alone group, NCC group and CC group detected by western blot. **D** Quantitative statistics of protein levels in (**C**). **E** Correlation analysis of BNIP3 expression and AMPK signaling pathway in TCGA-LIHC cohort. **F** As in (**E**) but in ICGC-LIHC cohort. **G** Flow cytometry analysis of 2-NBDG-FITC-A and cell proportion at 48 h after cell attachment in Huh7R (Alone group) /CCHuh7R (CC group) treated with AMPK-activator-2, AMPK-IN-3 or AMPK-IN-3 + AMPK-activator-2. **H** Mountain map of 2-NBDG-FITC-A in (**G**). * CCHuh7R+AMPK-activator-2 vs CCHuh7R-Control (*p* < 0.05); # CCHuh7R-AMPK-IN-3 vs CCHuh7R-Control (*p* < 0.05); & CCHuh7R+AMPK-IN-3 + AMPK-activator-2 vs CCHuh7R+AMPK-IN-3 (*p* < 0.05); $ Huh7R-AMPK-activator-2 vs Huh7R-Control (*p* < 0.05); ¥ Huh7R+AMPK-IN-3 vs Huh7R-Control (*p* < 0.05); % Huh7R+AMPK-IN-3 + AMPK-activator-2 vs Huh7R+AMPK-IN-3 (*p* < 0.05). **I** Quantitative statistics of ratio m + /m- in (**G**). **J** Cellular lactic acid production levels at 48 h after cell attachment in Huh7R (Alone group) /CCHuh7R (CC group) treated with AMPK-activator-2, AMPK-IN-3 or AMPK-IN-3 + AMPK-activator-2 by flow cytometry cell sorting. **K** (Top) High content immunofluorescence imaging of colocalization of autophagosomes (Cy5.5-LC3: purple) and mitochondria (TOMM20: green) at 48 h after cell attachment in Huh7R (Alone group) /CCHuh7R (CC group) treated with AMPK-activator-2 using 63X water immersion objective lens. (Bottom) Quantification of colocalization between LC3 (purple peak) and TOMM20 (green peak) in the above groups. Purple/green peak height represents fluorescence intensity of LC3B/TOMM20; overlapping peaks indicate colocalization numbers between LC3B and Tomm20. **L** Protein levels of AMPK, p-AMPK and ENO2 at 48 h after cell attachment in Huh7R (Alone group) /CCHuh7R (CC group) treated with AMPK-activator-2, sh*ENO2* or sh*ENO2* + AMPK-activator-2 detected by western blot. **M** Quantitative statistics of protein levels in (**L**). **N** Protein levels of AMPK, p-AMPK and BNIP3 at 48 h after cell attachment in Huh7R (Alone group) /CCHuh7R (CC group) treated with oe*BNIP3*, AMPK-IN-3 or oe*BNIP3* + AMPK-IN-3 detected by western blot. **O** Quantitative statistics of protein levels in (**N**). Three independent experiments were conducted, and the values are represented by means ± SEM using a two-way ANOVA with Tukey’s multiple comparisons test (**D**, **H**, **J**, **M** and **O**) or Ordinary one-way ANOVA with Sidak’s multiple comparisons test (*I*). *p < 0.05, #*p* < 0.05, $*p* < 0.05, ¥*p* < 0.05, &*p* < 0.05, %*p* < 0.05, ns = non-statistically significant. See also Fig. S6.

**Fig. 7:**
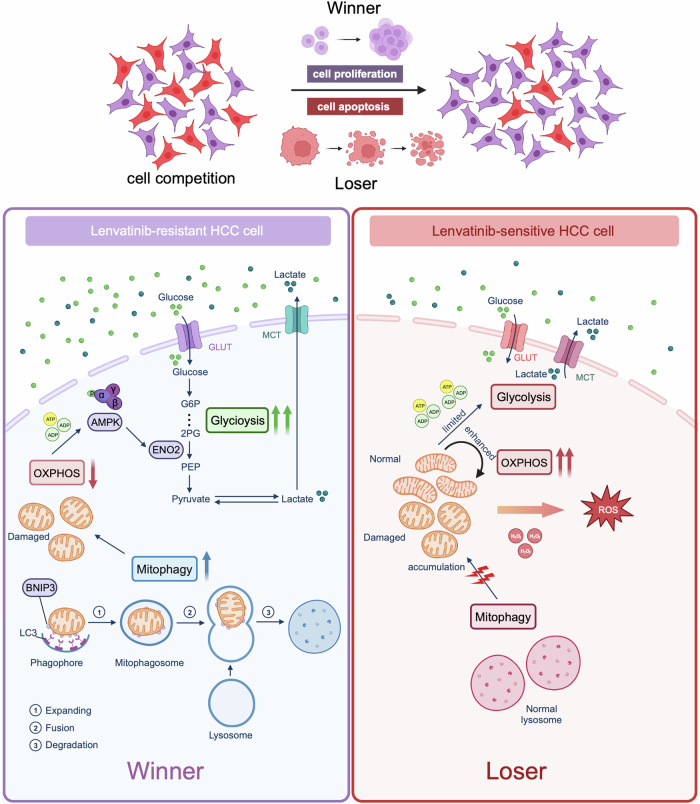
Cell competition mechanism between lenvatinib-resistant cell (Winner) and lenvatinib-sensitive cell (Loser) in HCC. In HCC coculture system, overexpressed BNIP3 activates mitophagy activity through binding with LC3, which eliminates damaged mitochondria of lenvatinib-resistant HCC cell; mitochondria reduction results in the decrease of oxidative phosphorylation (OXPHOS) levels; subsequently, energy imbalance signal (ADP/ATP) caused by weaken OXPHOS triggers the phosphorylation of AMPK sensor; enabled AMPK specifically targets ENO2 and therefore endows resistant cell with enhanced glycolysis, which facilitates the competitive and grabbing abilities for glucose from environment and opponent, eventually boosting cell proliferation. In lenvatinib-sensitive HCC cell, this failed mechanism by which BNIP3 modulates energy metabolism shifting continually activates and overuses mitochondrial OXPHOS for energy compensation, which aggravates damaged mitochondria accumulation and ROS production; limited metabolism function and oxidative pressure disqualify lenvatinib-sensitive HCC cell’s competition for resource, which eventually induce cell apoptosis.

Our findings above implied that ENO2 is a specific target in multiple potential proteins of glycolysis which was heightened by BNIP3-mediated mitophagy in cell competition scenario. However, in the stressful and nutritionally competitive environment, the logically biological relevance between resistant and sensitive HCC cells or whether there exists an inductor to convey the information caused by energy metabolism changes is still unclear. Of note, besides glycolysis pathway, up-regulated AMPK signaling pathway was also enriched in GSEA analyses for confirming the relationship between BNIP3 and glycolysis pathway in two clinical cohorts (Fig. 6A, B) and evidenced by western blot analysis (Fig. 6C, D; Supplemental Fig. S7A, B). In addition, ssGSEA analysis also showed that there existed a remarkably positive correlation between BNIP3 expression and AMPK signaling pathway or AMPK expression (catalytic subunit: PRKAA1, PRKAA2; non-catalytic subunit: PRKAB1) (Fig. 6E, F; Supplemental Fig. S7C, D). As is well-known, AMPK pathway participates in cellular energy sensing and provokes glucose uptake through heterotrimeric kinase complex [36]. Hence, we speculated the BNIP3-AMPK-ENO2 crosstalk exists and may function as the regulator of glycolytic metabolism and promoter of competitive advantage phenotypes of lenvatinib-resistant HCC cells in cell competition scenario. With regard to this, we first assessed the effect of AMPK on glycolysis and competition behavior of resistant HCC cells. Consistent with BNIP3’s effects, AMPK inhibition (AMPK-IN-3) contributed to the reduction of glycolysis levels and competitive advantage of CCHuh7R/CCPLC-PRF-5R over the control, which was rescued by the treatment of AMPK activator (AMPK-activator-2) (Fig. 6G–J; Supplemental Fig. S7E–H). These results provided the evidence that AMPK might serve as an inductor for modulating glycolysis and competitive dominance of CCHuh7R/ CCPLC-PRF-5R cells in cell competition scenario. Furthermore, we sought to systematically investigate the existence of the BNIP3-AMPK-ENO2 axis and its regulatory relationship in our competitive models. As expected, compared with corresponding control groups, activated AMPK not only increased ENO2 expression levels of co-cultured resistant HCC cells, (Fig. 6L, M; Supplemental Fig. S7I) but also attenuated the inhibitory effects of CCHuh7R-sh*ENO2* on the above glycolysis indicators (excepting unchanged AMPK protein expression level) and cell competition phenomenon (Supplemental Fig. S7J–M), suggesting that AMPK-regulated glycolysis and competitive dominance of CCHuh7R/CCPLC-PRF-5R required the existence of ENO2. On the other hand, overexpressed BNIP3 up-regulated the level of phosphor-AMPK while no significant alteration was found in the expression levels of BNIP3 and the colocalization of mitophagy-related proteins of CCHuh7R/CCPLC-PRF-5R under AMPK inhibition or activation treatment (Fig. 6K, N and O**;** Supplemental Fig. S7N). Nevertheless, overexpressed BNIP3 up-regulated the level of phosphor-AMPK (Fig. 6N, O**;** Supplemental Fig. S7N) and elevated AMPK-IN-3-inhibited metabolic levels and enhanced the outcompeting of CCHuh7R cells in cell competition scenario (Supplemental Fig. S7O–R). Overall, our data indicated that mitophagy facilitates glycolysis via BNIP3-AMPK-ENO2 crosstalk for persistently maintaining the competitive advantage of lenvatinib-resistant HCC cells in cell competition scenario, which comprehensively deciphered in Fig. 7.

## Discussion

Despite the advances in targeted drugs and treatment strategies for advanced HCC such as lenvatinib, cabozantinib, and multidrug combination strategy, the clinical benefit remains dismal owing to the occurrence of acquired chemoresistance [37–39]. At present, the molecular mechanism of chemoresistance in HCC has been widely explored and demonstrated; the formulation of overcoming chemoresistance strategies mainly relies on biological foundations and relevant treatment tactics including the log kill method, synthetic lethal method and immunotherapies [5–7] are in practice. Unfortunately, the treatment tactics against tumor heterogeneity have not been fully considered. Tumor heterogeneity closely resembles a Gordian Knot, where epigenetic changes and clonal evolution endow it with volatile phenotypes [40], exploring drug resistance reversal strategies against tumor heterogeneity might be a significantly promising approach. In recent years, increasing studies have reported that a heterogeneity-based biological phenotype, the supervision and exclusion of less suited “loser” cells by nearby “winner” cells, termed cell competition, is closely linked to tumorigenesis and development [41–43]. Importantly, a variety of cases and models have demonstrated that tumor cells with higher competence can constantly maintain and spread the most aggressive characteristics to outcompete relatively less competent cells [44], which precisely conforms to the feature of resistant tumor cells [19, 20]. Thus, this evidence indicates that cell competition is expected to become a potential biological basis for tumor treatment or overcoming drug resistance.

In this study, through constructing a mimetic lenvatinib-resistant-related HCC cell competition model, we first provided an evidence that there exists cell competition between lenvatinib-resistant cells and lenvatinib-sensitive cells in HCC. During this process, lenvatinib-resistant cells obtain competitive advantage based on innate sub-foundation and further aggregate their proliferation characteristic at the price of lenvatinib-sensitive cells through energy metabolism reprogramming. It is reported that drug-resistant cells generally have superior capacities of proliferation, metastasis and metabolism [19, 20], which is in line with the verified characteristic of the winner as well as the reason for its triumph [45, 46]. These results indicate the emergence of drug resistance potentially may result from the fittest survival of resistant cells over primary cells during clinical treatments, and prove the study value of overcoming drug resistance against cell competition. Nevertheless, it is imperative to elucidate their detailed mechanisms which remain largely imprecise.

Recent studies have manifested that activated glycolysis or energy metabolism differences are required to drive cell competition [47]. Of note, our transcriptome analyses showed that, in HCC cell competition scenario, lenvatinib-resistant cells captured the increased glycolysis activity but the attenuated oxidative phosphorylation level as well as decreased mitochondria mass; however, lenvatinib-sensitive cells obtain opposite metabolic features. Importantly, results from in-vitro experiments including elevated glucose uptake and lactic acid secretion, high-expressed glycolysis-related key enzymes, low expression of rate-limiting enzymes in oxidative phosphorylation, reduced mitochondrial membrane potential and decreased mitochondrial mass in lenvatinib-resistant HCC cells (CCHuh7R) re-confirmed these phenotypes above; furthermore, low glucose treatment exhibited a decreased glucose uptake and attenuated competitive advantage of lenvatinib-resistant HCC cells (CCHuh7R), suggesting that glucose availability also has an effect upon winner’s glycolysis and cell competition; in contrast, oxidative phosphorylation agonist Calcitriol presented no significant effect on glucose uptake but notably exacerbated cell competition through promoting opponent’s death. These results imply that metabolic differences exist between lenvatinib-resistant HCC cells (CCHuh7R) and sensitive HCC cells (CCHuh7m), which finally leads to their distinct competition fitness. As we know, mitochondrial oxidative phosphorylation and glycolysis are crosslinked processes [21] and tumor cells grab enough nutrients for survival under various pressures through transforming into glycolysis following the Warburg effect [22, 23]. Accordingly, it is reasonable to infer that lenvatinib-resistant HCC cells competitively could obtain sufficient glucose substrates for survival and continually maintain their winner status through intelligently switching energy metabolism from mitochondrial oxidative phosphorylation to principal glycolysis under the stressful cell competition environment. However, although the decreased mitochondrial membrane potential caused by energy metabolism reprogramming is an appropriate way to maximize survival, the reason why that occurred and aggregated by Calcitriol in lenvatinib-sensitive HCC cells (CCHuh7R), which is contrary to its fate, remains unclear. It is possible that sensitive HCC cells (CChuh7m) confront certain oxidative stress such as elevated ROS levels in the cell competition scenario [48, 49]. In addition, since the death forms of loser cells were no longer confined to apoptosis, but have presented different codes such as necroptosis, autophagy, entosis, cell-independent extrusion and cell senescence in cell competition [50–55], the specific death form of lenvatinib-sensitive cells is required to be clarified in the future.

In recent decades, multi-omics analyses and numerous studies have indicated that mitophagy not only facilitates multiple malignance behaviors [56–58] and tumor heterogeneity [59], but also induces resistance to anti-cancer drugs including doxorubicin, cisplatin, sorafenib and lenvatinib via various pathways and core molecules such as ARIHI, PINK1, NIX and BNIP3 [30, 60–62]. Crucially, mitochondrial homeostasis and glycolysis activities including glucose uptake and lactic acid production also are regulated by mitophagy-related proteins [27, 28, 63]. However, the mechanisms in mitophagy, drug resistance and cell competition hitherto remain fully unknown. In this study, results from a variety of molecular experiments combined with transcriptome analyses imply that BNIP3-mediated mitophagy induces energy metabolism reprogramming and competitive fitness of lenvatinib-resistant HCC cells (CCHuh7R/CCPLC-PRF-5R) in cell competition scenario. Additionally, it was confirmed that based on BNIP3 knockdown, reduced glycolysis activities and decreased expressions of related proteins, especially key enzyme ENO2, and alleviated competitive advantage, which can be rescued by the transient overexpression of BNIP3. These results not only further support our conclusion, but also suggest the effect of mitophagy on glycolysis in lenvatinib-resistant HCC cells (CCHuh7R/CCPLC-PRF-5R) is targeted by ENO2, which has been verified in various cancers [33, 34]. Although subsequent results under dual gene modification of ENO2 and BNIP3 further validate and reveal the terminal target of ENO2, we still don’t understand why BNIP3-mediated mitophagy is specifically associated with ENO2 and importantly, how to connect with them in lenvatinib-resistant HCC cells (CCHuh7R/CCPLC-PRF-5R). As is well-known, under the limited source or stressful environment, metabolic alteration or reprogramming is bound to induce energy imbalance, and according to studies, the metabolic sensor AMPK subsequently is activated resulting from the rise of AMP/ATP ratio, eventually targeting glycolytic enzyme [64]. Therefore, results including significantly enriched AMPK pathway, high-expressed phosphorylated AMPK and AMPK-IN-3 inhibitor-induced inhibition in glycolysis activities and competitive advantage of lenvetinib-resistant HCC cells (CCHuh7R/CCPLC-PRF-5R) indeed prove that AMPK serving as signal sensor plays an essential role in enhancing glycolysis and maintaining winner status of lenvetinib-resistant HCC cells (CCHuh7R/CCPLC-PRF-5R) in cell competition scenario. Moreover, increased ENO2 expression and attenuated inhibitory effects of ENO2 knockdown on glycolysis activities under AMPK activation, unchanged BNIP3 expression and mitophagy colocalization under AMPK inhibition or activation, decreased inhibitory influence of AMPK-IN-3 on AMPK expression and glycolysis activities and population advantage under BNIP3 overexpression and previous BNIP3-ENO2 regulatory axis first reveal a vital role for BNIP3-AMPK-ENO2 crosstalk in continuously maintaining competitive advantage of lenvatinib-resistant HCC cells (CCHuh7R/CCPLC-PRF-5R). Of note, it has been reported that AMPK modulates energy and metabolic homeostasis through phosphorylating key glycolytic enzyme PFKFB3 and, on the other hand, AMPK-induced recycle signal can be effectively activated to devour cell debris and then promote the growth of lung cancer [65]; therefore, the detailed functions of AMPK-ENO2 axis and the effects of AMPK on lenvatinib-sensitive HCC cells will be clarified in the future.

Furthermore, a growing body of works suggests that targeting cell competition may obtain a favorable therapeutic efficacy. COX-2/PGE_2_ inhibitor can facilitate apical extrusion of RasV12-transformed cells by maintaining a process termed epithelial defense against cancer (EDAC) and finally suppress tumor initiation [66]. In addition, lithium effectively eliminates the competitive advantage of Apc-mutant ISCs by promoting the WNT pathway in normal ISCs, eventually preventing the formation of adenomas [14]. Given the molecular mechanism elucidated firstly in the study, we also assessed the value of inhibiting BNIP3, a critical protein in promoting competition advantage of lenvatinib-resistant HCC cells (CCHuh7R/CCPLC-PRF-5R) in cell competition scenario, in a xenografted tumor model and verified that BNIP3 inhibition significantly sensitized the anti-tumor efficacy of lenvatinib in HCC. The result enhanced our broader understanding of the relationship between cell competition and drug resistance in HCC.

In summary, our study reported that mitophagy can profoundly boost shifting energy production from mitochondrial oxidative phosphorylation to glycolysis via BNIP3-AMPK-ENO2 signaling, thereby maintaining lenvatinib-resistant HCC cells’ growth and leading to HCC development through sacrificing sensitive cells in cell competition scenario. However, based on the clinical universality of muti-drug combination therapy, the mechanism of BNIP3-AMPK-ENO2 axis in HCC will need to be verified in future clinical studies to definitively link cell competition with HCC lenvatinib resistance.

As cell competition driven by energy metabolism reprogramming is notoriously involved in tumorigenesis and progression, and increasing evidence suggests a pivotal role for metabolic shifting-mitophagy in facilitating drug resistance and a variety of malignant behaviors [57–59], the knowledge gained will provide a foundation for understanding that cell competition serving as an essential contributor of tumor heterogeneity, is expected to become a potential therapeutic biophysical property, and targeting mitophagy-related BNIP3 can be a promising strategy for overcoming drug resistance in HCC, even other diverse tumors.

## Materials and methods

### Cell lines

Human liver tumor cell lines (Huh7, PLC-PRF-5) obtained from the Liver Cancer Institute, Zhongshan Hospital, Fudan University (Shanghai, China) were cultured in High glucose DMEM (Thermo Fisher, USA) with 10% fetal bovine serum (SORFA, China), 1% penicillin and streptomycin (Beyotime China). The lenvatinib-resistant cells (Huh7R) were established from Huh7 cells by exposure to gradually increasing concentrations of Lenvatinib (MCE, USA). The final highest concentration and maintenance dose of Huh7R were 30 μm and 20 μm, respectively. Lenvatinib-resistant cells (PLC-PRF-5R) were obtained from Ke Lab, Zhongshan Hospital, Fudan University (Shanghai, China). To track or differentiate cells, Huh7/PLC-PRF-5 stably expressing mcherry (Huh7m/ PLC-PRF-5m) were constructed via transfecting Huh7/PLC-PRF-5 with CMV-MCS-EF1a-mCherry-T2A (Genechem, China) and following the selection of DMEM containing 5ug/ml puromycin (Beyotime, China). All cells were maintained at 37 °C with 5% CO2.

### Mice

Six-week-old female BALB/c nude mice purchased from the Chinese Academy of Medical Sciences (Beijing, China) were housed and raised at the laboratory animal center of Zhongshan Hospital, Fudan University (Shanghai, China), following the guidelines of biosafety and bioethics. All mice were provided with standard laboratory chow and accommodated within a controlled environment maintained at 22 °C, following a 12 h light-dark cycle. For the subcutaneous model, total 1 × 10^7^ cells of single Huh7R/PLC-PRF-5R, NCC group (NCCHuh7m mixed with NCCHuh7, NCCPLC-PRF-5m mixed with NCCPLC-PRF-5, at 1:1 ratio), or CC group (CCHuh7m mixed with CCHuh7R, NCCPLC-PRF-5m mixed with NCCPLC-PRF-5R, at 1:1 ratio) were injected subcutaneously to the armpits of five nude mice per group. All BALB/c mice were monitored and those tumor-carried BALB/c mice were assigned to the groups as described above. For the cell population analysis of subcutaneous model, mice (NCC group, CC group) were sacrificed after 2 weeks and their tumor tissues were dissected and administered by H&E staining before fluorescence microscope imaging and quantification analysis. For the cell population analysis and tumor growth analysis of orthotopic model, subcutaneous tumors of mice (Huh7R group, CC group) were collected while reaching 1 cm in diameter and cut into pieces (1 mm^3^) under aseptic conditions; subsequently, fresh subcutaneous tumor pieces were orthotopically implanted in the liver of five healthy mice per group. After 2 weeks, mice were sacrificed to harvest liver orthotopic tumors for calculating tumor volume [width (mm) × width (mm) × length (mm)/2] and following the imaging experiment described above. For the effect validation of combination therapy, subcutaneous mice models (CC group) were treated with vehicle (sterile distilled water) or lenvatinib (10 mg/kg) with or without BNIP3 inhibitor olomoucine (6 mg/kg; CaymanChemical, USA) by intraperitoneal (I.P.) on days 7, 10, 12 and 14 after tumor inoculation; on day 21, mice were sacrificed and their tumors were harvested for volume analysis and administered by H&E staining before fluorescence microscope imaging and quantification analysis. All the tumor data from the above group were processed by ImageJ quantification analyses.

### Cell competition assay

To visually observe cell competition behavior in vitro, Huh7/Huh7R were cocultured with Huh7m (NCC group, CC group) at 1:1 ratio, or Huh7R, Huh7 and Huh7m (Alone group) were cultured alone overnight. After cell attachment, a time-lapse observation experiment was executed for 48 h in 1 ml medium per well by high content imaging system (Perkin Elmer Operetta). Images were exposed for 20 ms in the mcherry channel and captured every 30 min for 48 h using a 10X/20X objective lens. For flow cytometry cell competition analyses, Huh7/Huh7R were cocultured with Huh7m (NCC group, CC group), PLC-PRF-5/PLC-PRF-5R were cocultured with PLC-PRF-5m (NCC group, CC group) at 1:1 ratio, or Huh7R/PLC-PRF-5R and Huh7m/PLC-PRF-5m (Alone group) were cultured alone. After adherence, cells were collected at 24 h/48 h through trypsin (Thermo Fisher, USA) digestion and 1X PBS (Thermo Fisher, USA) resuspension. Specifically, Alone groups were manipulated as follows for comparison: collected Huh7R/PLC-PRF-5R cells were mixed with collected Huh7m/PLC-PRF-5m cells. All cells were measured by BD FACSCanto II (BD Biosciences). The experiments were carried out independently three times.

### shRNA/plasmid transfection and chemicals treatment

To analyze the molecular mechanism of cell competition, Huh7R/PLC-PRF-5R stably expressing *BNIP3*/*ENO2*-short hairpin RNA (sh*BNIP3*/*ENO2*) was constructed via transfecting cells with sh*BNIP3*/*ENO2* lentivirus obtained from the Liver Cancer Institute and following the selection of DMEM containing 5 ug/ml puromycin; Huh7R/PLC-PRF-5R transitorily overexpressing *BNIP3* (oe*BNIP3*) was constructed via transfecting Lipofectamine 3000 reagent (Thermo Fisher, USA) with *BNIP3* plasmid (Sangon, China) and following the selection of DMEM containing 5 ug/ml neomyein sulfate (Solarbio, China); AMPK-activated/inhibited Huh7R/PLC-PRF-5R was constructed by pre-treating with 2 μm AMPK-activator-2 (MCE, USA)/15μm AMPK-IN-3 (MCE, USA). For rescue assays, Huh7R-sh*BNIP3*/PLC-PRF-5R-sh*BNIP3* was transitorily transfected Lipofectamine 3000 reagent with *BNIP3* expression plasmid and following the selection of DMEM containing 5 ug/ml neomycin sulfate; Huh7R-sh*ENO2*/PLC-PRF-5R-sh*ENO2* was transitorily transfected Lipofectamine 3000 reagent with *ENO2* expression plasmid (Sangon, China) and following the selection of DMEM containing 5 ug/ml neomycin sulfate, or treated with AMPK-activator-2; Huh7R-oe*BNIP3*/PLC-PRF-5R-oe*BNIP3* was treated with AMPK-IN-3; Huh7R/PLC-PRF-5R were pre-treated with AMPK-activator-2 before adding AMPK-IN-3; then all the above Huh7R/PLC-PRF-5R cells were cultured alone or cocultured with CCHuh7m/CCPLC-PRF-5m for 48 h before performing corresponding assays. To validate the metabolic difference of CCHuh7R and CCHuh7m, CC group was treated with low glucose addition (Thermo Fisher, USA)/ 12.5 μg/mL Calcitriol (Aladdin, China) and then performed corresponding assays. Mycoplasma removal agent (Beyotime, China) was regularly applied to all cell lines.

The shRNA/plasmid target sequences are listed in supplementary Table 1.

### Cytotoxicity experiment

To further observe the drug sensitivity between Huh7R and Huh7, Huh7/Huh7R (1 × 10^4^ cells/100ul) were plated into 96-well plates at 100 ul/well and treated with lenvatinib at various concentrations (0, 0.01, 0.1, 0.5, 1, 10, 20, 40 and 100 μm) for 48 h after attachment. Subsequently, the medium was removed, and cells were incubated in the fresh medium mixed with CCK8 working fluid (Beyotime, China) for 1 h at 37 °C. Cell viability was measured through detecting the absorbance at wavelengths of 450 nm using microplate reader (BioTek, USA). The experiments were carried out independently three times.

### Cell proliferation assay

To compare the short-term proliferative capacity of Huh7 and Huh7m, Huh7 (3 × 10^3^) and Huh7m (3 × 10^3^) were respectively seeded into 96-well plates for 24 h/48 h. Next, the complete media was removed, and cells were incubated in the fresh medium mixed with CCK8 working fluid for 1 h at 37 °C after washing by 1X PBS. Cell viability was measured through detecting the absorbance at wavelengths of 450 nm using microplate reader. The experiments were carried out independently three times.

### Colony formation assay

To compare the long-term proliferative capacity of Huh7 and Huh7m, Huh7 (1 × 10^3^) and Huh7m (1 × 10^3^) were separately seeded into 12-well plates for 15 days. In the meantime, media was changed every five days after 1X PBS washing. Lastly, cells were fixed in 4% paraformaldehyde (Biosharp, China) and stained with crystal violet staining solution (Beyotime, China). Afterwards, all the colonies were counted and quantitative analyses. The experiments were carried out independently three times.

### ROS measurement

To measure the intracellular ROS level between Huh7 and Huh7m, Huh7 (3 × 10^5^) and Huh7m (3 × 10^5^) were respectively seeded into 12-well plates overnight. After adherence, cells were followed by trypsinization after staining with 5 µm 2, 7-dichlorofluorescein diacetate (DCFH-DA; Beyotime, China) for 30 min at 37 °C (protect from degradation of light). Subsequently, cells were resuspended by 1X PBS resuspension and collected. DCFH-DA intensity was detected by the FITC-A channel using BD FACSCanto II (BD Biosciences). The experiments were carried out independently three times.

### Early apoptosis assay

To contrast the early apoptosis in Huh7 and Huh7m, Huh7 (3 × 10^5^) and Huh7m (3 × 10^5^) were seeded into 12-well plates overnight. After adherence, cells were followed by trypsinization after staining with Annexin V-FITC binding buffer and Annexin V-FITC (Beyotime, China) for 20 min at 37 °C (protect from degradation of light). Subsequently, cells were resuspended by 1X PBS resuspension and collected. Annexin V intensity was detected by the FITC-A channel using BD FACSCanto II (BD Biosciences). The experiments were carried out independently three times.

### 2-NBDG glucose uptake assay

Cells mentioned previously were cultured for 48 h and stained with 100ul glucose-free medium (Thermo Fisher, USA) containing 200μm 2-NBDG (MKBio, China) and incubated at 37 °C for 80 min (protect from degradation of light). For flow cytometric analysis (BD Biosciences), cells were digested and resuspended by 1X PBS resuspension. 2-NBDG intensity was detected by the FITC-A channel. The above experiments were carried out independently three times.

### Lactic acid detection assay

Cells mentioned previously were cultured for 48 h; cells of coculture groups were divided by flow sorting (BD Biosciences) based on mcherry fluorescence expression. Afterwards, cells of each group were washed with 1X cold PBS and resuspended by 4X Assay Buffer XII (Abcam, UK) followed by a full homogenization. After centrifuging for 5 min at 4 °C, the supernatant was collected and seeded into 96-well plates; then the working mixture solution (Abcam, UK) was added into sample wells and incubated at room temperature for 30 min. The lactic acid concentration was measured by detecting the absorbance at wavelengths of 450 nm using a microplate reader. The experiments were carried out independently three times.

### Mitochondrial membrane potential assay

Cells mentioned previously were cultured for 48 h and stained with 0.1X Rhodamine123 (Beyotime, China) and incubated at 37 °C for 30 min (protect from degradation of light). For flow cytometric analysis (BD Biosciences), cells were digested and resuspended by 1X PBS resuspension. Rhodamine123 intensity was detected by the FITC-A channel. The above experiments were carried out independently three times.

### Dead cell detection assay

Cells mentioned previously were cultured for 48 h and followed by trypsinization after staining with SYTOX Green (Thermo Fisher, USA) and incubating at 37 °C for 30 min (protect from degradation of light). Subsequently, cells were resuspended by 1X PBS resuspension and collected. SYTOX Green intensity was detected by the FITC-A channel using BD FACSCanto II (BD Biosciences). The experiments were carried out independently three times.

### Mitochondrial morphology assay

Cells mentioned previously were cultured for 48 h and stained with 1:1000 Mito-Tracker Green (Beyotime, China) and incubated at 37 °C for 90 min (protect from degradation of light). Two cellular samples were prepared for different experiments. For high content fluorescence imaging (Perkin Elmer Operetta), cells were also stained with and 1X DAPI for 10 min and added in 1X PBS after removing the mixture and analyzed by the Harmony software (Perkin Elmer) using a 63X water immersion objective lens. For flow cytometric analysis (BD Biosciences), cells were digested and resuspended by 1X PBS resuspension. The above experiments were carried out independently three times.

### Transmission electron microscope (TEM)

Cells mentioned previously were cultured for 48 h; cells of coculture groups were divided by flow sorting (BD Biosciences) based on mcherry fluorescence expression. Subsequently, cells were completely fixed with 4% paraformaldehyde and 2% glutaraldehyde for 1 h following post-fixing in 1% osmium tetroxide and 0.5% potassium ferricyanide. Lastly, samples were embedded in resin at 80 °C for 24 h. Images were captured via using the transmission electron microscope.

### Western blot

Cells mentioned previously were cultured for 48 h; then cells of coculture groups were divided by flow sorting (BD Biosciences) based on mcherry fluorescence expression. Afterwards, cells of all groups were separately lysed in 1X RIPA cell lysis buffer containing 1X protease and phosphatase inhibitors (Beyotime, China). BCA assay was then performed to determine collected protein concentrations. A SDS-PAGE loading buffer was added to our samples and heated at 100 °C for 5 min. In the next step, samples were executed for 10% SDS PAGE and western transfer. Subsequent 1 h block by western sealing buffer and primary antibodies were performed at 4 °C overnight. Primary antibodies utilized in this paper were including β-Actin (1:10000, #AC038, ABclonal, USA), EGFR (1:1000, #A23452, ABclonal, USA), p-EGFR (1:500, #AP0992, ABclonal, USA), VEGFR2 (1:500, #A5609, ABclonal, USA), p-VEGFR2 (1:500, #AP1385, ABclonal, USA), ENO2 (1:1000, #A19091, ABclonal, USA), GLUT1 (1:1000, #A11208, ABclonal, USA), HK1 (1:1000, #A0533, ABclonal, USA), HK2 (1:1000, #A2867S, Cell signaling technology, USA), MCT1 (1:1000, #A3013, ABclonal, USA), MCT4 (1:1000, #A3016, ABclonal, USA), PFKP (1:1000, #A12160, ABclonal, USA), PKM2 (1:1000, #A20991, ABclonal, USA), LDHA (1:1000, #3582 S, Cell signaling technology, USA), LDHB (1:1000, #A7625, ABclonal, USA), GAPDH (1:1000, #A19056, ABclonal, USA), LC3B (1:1000, #AP1802A, Abcepta, CHN), TOMM20 (1:1000, #42406 S, Cell signaling technology, USA), BNIP3 (1:1000, #A19593, ABclonal, USA), AMPK (1:1000, #5831 S, Cell signaling technology, USA), p-AMPK (1:1000, #AP1002, ABclonal, USA), IDH3A (1:1000, #15909-1-AP, Proteintech, USA), COX7A (1:1000, #11413-1-AP, Proteintech, USA), SDHB (1:1000, #10620-1-AP, Proteintech, USA), SDHA (1:1000, #,14865-1-AP, Proteintech, USA), UQCRC2 (1:1000, #67547-1-Ig, Proteintech, USA), OGDH (1:1000, #15212-1-AP, Proteintech, USA) and CS (1:1000, #67784-1-Ig, Proteintech, USA). After washing 3 times by TBST buffer, membranes were incubated at 37 °C for 1 h by HRP-conjugated goat anti-rabbit-IgG (1:1000, #7074, Cell signaling) or HRP-conjugated horse anti-mouse-IgG (1:1000, #7076, Cell signaling). At last, chemiluminescence was used for detecting protein levels of membranes. The above experiments were carried out independently three times.

### Mitophagy colocalization assay

Cells mentioned previously were cultured for 48 h and fixed in 4% paraformaldehyde and then permeabilized in 0.5% Triton X-100 for 15 min to improve antigen accessibility. After permeabilization, 30 min block by 5% BSA and primary antibodies specific for LC3B, TOMM20 were performed at 4 °C overnight. After washing 3 times by PBST buffer, cells were incubated at 37 °C for 1 h by secondary antibodies including Cy5.5 goat anti-mouse IgG (Abcam, UK) and Alexa fluor 488 goat anti-rabbit IgG (Abcam, UK) (protect from degradation of light). At last, cells were analyzed by the Harmony software (Perkin Elmer) using a 63X water immersion objective lens after washing by TBST buffer. The above experiments were carried out independently three times.

### RNA isolation and library preparation

Huh7R/Huh7 and Huh7m were cocultured in a 1:1 ratio for 48 h. Flow sorting (BD Biosciences) was used to separate the different cell populations in the coculture sample based on mcherry expression. Three biological replicates for each of the different culture conditions of Huh7, Huh7m and Huh7R were performed. Equivalent cells were collected, and total RNA of the cells was extracted by TRIzol reagent (Thermo Fisher, USA) conducting the same method in RT-qPCR experiment. A total of 2 µg RNA per specimen was ready for RNA sequencing experiment. Agilent 2100 Bioanalyzer (ABI, USA) was performed to assess the quantity and quality of the cDNA libraries. The Double-stranded PCR products were treated with thermal denaturation and cyclization reaction using the splint oligo sequence and the final library was generated. Phi29 (Thermo Fisher, USA) was utilized for amplifying the single-stranded circular DNA and then a DNA nanoball was generated.

### RNA-seq and differential expression analysis

MGISEQ-2000 platform was used to sequence the prepared libraries mentioned above. SOAPnuke [67] (Version 1.5.2) was performed to clean the sequencing data via removing reads that contained sequencing adapter, contained >20% of low-quality base ratio (ratio less than or equal to 5) and contained >5% unknown base (‘N’ base) ratio. The cleaned reads, consequently, were acquired and stored in FASTQ format. Then, HISAT2 [68] was used to align the cleaned reads to the reference human genome. Bowtie2 [69] (Version 2.2.5) was used to align the cleaned reads to the reference coding gene set. Afterwards, RSEM [70] (Version 1.2.12) was used to compute the expression level of gene and Count data was generated. Essentially, differential expression analysis (DEA) for Count data was executed using the Limma-Voom algorithm of R-package Limma (Version 3.52.1). Selection criteria of significant differentially expressed genes (DEGs) was as follows: *p*-value < 0.05, FDR < 0.05 and |Log2FC | > 1. Transcripts per million (TPM) normalization was used to scale expression per transcriptome.

### Data resource and analysis

RNA-seq data of 374 HCC samples were obtained from The Cancer Genome Atlas-Liver Hepatocellular Carcinoma (TCGA-LIHC) cohort downloaded from the UCSC Xena website (http://xena.ucsc.edu/). RNA-seq data of 240 HCC samples were acquired from International Cancer Genome Consortium-Liver Hepatocellular Carcinoma (ICGC-LIHC) cohort. The annotation information data (gencode.v22.annotation.gene.probeMap) downloaded from UCSC Xena website was performed to match the Ensemble Gene IDs to Gene Symbols. According to the median value of BNIP3 mRNA expression, TCGA-LIHC cohort and ICGC-LIHC cohort were respectively divided into BNIP3 high-expression group and BNIP3 low-expression group. Afterwards, differential expression analysis, Pearson correlation analysis and Gene set enrichment analysis were executed in TCGA-LIHC cohort and ICGC-LIHC cohort, respectively.

### Gene set enrichment analysis (GSEA)

To explore biological characteristics in indicated comparative groups respectively, GSEA was performed to score the gene sets based on the DEA via using R-package clusterProfiler (Version 4.4.2). Genes rank in the indicated gene sets was utilized in accordance with results of differential expression analysis within CCHuh7R vs Huh7R group, CCHuh7R vs NCCHuh7 group, Huh7R vs Huh7 group and BNIP3 high-expression vs BNIP3 low-expression group in two cohorts mentioned above. Customized gene set composed by 38 glycolysis-related pathways based on KEGG database (http://www.genome.jp/kegg/) was performed to explore the regulatory mechanism between Mitophagy and Glycolysis in our indicated groups. Selection criteria of significant biological characteristics was as follows: *p*-value < 0.05, FDR < 0.05 and |NES | > 1.

### Gene set variation analysis (GSVA)

To investigate the mitochondria mass and activities in indicated comparative groups respectively, GSVA was carried out on log2 (normalized TPM + 1) expression values within CCHuh7R vs Huh7R group and CCHuh7R vs NCCHuh7 group via using R-package GSVA (Version 1.44.1). Customized gene set composed by pathways of mitochondria mass and activities based on GO database (http://geneontology.org/page/go-database) was performed to explore indicated phenotypes. GSVA enrichment score was generated for each sample and pathway in indicated groups. Afterwards, DEA was executed and, selection criteria of significant differentially activities were as follows: *p*-value < 0.05, FDR < 0.05.

### Single sample GSEA (ssGSEA)

To examine the association of BNIP3 with certain pathways, ssGSEA was carried out on log2 (normalized TPM + 1) expression values within BNIP3 high expression vs BNIP3 low expression group in two cohorts mentioned above via using R-package GSVA (Version 1.44.1). The gene sets for AMPK signaling pathway and Glycolysis pathways were obtained in GSEA results based on customized gene set mentioned above. Enrichment score was generated for each sample in two cohorts, respectively. Afterwards, Pearson correlation analysis was implemented to determine correlation between BNIP3 expression and AMPK expression or two pathways described above via using R-package ggpmisc (Version 0.4.6).

### Data visualization statistics

Volcano plots were generated based on DEGs in indicated comparative groups. Heat maps were generated for indicated pathways-related DEGs, or pathways based on the results of GSEA and GSVA, or drug responses (Activity Area) in 81 LIMORE HCC cell models. UpSet plots were generated for Top 50 genes or up-regulated genes of glycolysis pathway based on DEGs and GSEA in indicated comparative groups via using R-package UpSetR (Version 1.4.0). Pearson’s correlation coefficient was conducted to determine the correlation between BNIP3 expression and AMPK expression, or two pathways described above in indicated groups, or correlation in 81 LIMORE HCC cell models under lenvatinib treatment. Visualization of analyses mentioned previously was plotted by using ggplot2 R package. All of analyses and visualizations were performed in the R software (Version 4.1.3) using multiple R packages. All the statistical analyses were performed by GraphPad Prism 9.0 software. The statistical significance was evaluated by *t*-test, ordinary one-way ANOVA with Sidak’s multiple comparisons test or two-way ANOVA with Tukey’s multiple comparisons test as described in Figure legends. *p < 0.05, #*p* < 0.05, $*p* < 0.05, ¥*p* < 0.05, &*p* < 0.05, %*p* < 0.05, ns/NS non-statistically significant. All data were represented as mean ± SEM.

### Supplementary information

supplementary files including supplementary figures, supplementary table 1 and figure legends

video1

video2

video3

video4

video5

video6

video7

video8

video9

video10

video11

video12

video13

full and uncropped WB images

## Data Availability

The access number of the processed expression datasets of RNA-seq from this paper is GSE235539. No original code was reported in this paper. Any other data in this paper are available upon reasonable request for the corresponding author.
