# Supplementary material for: BNIP3-mediated mitophagy boosts the competitive growth of Lenvatinib-resistant cells via energy metabolism reprogramming in HCC
Source: Cell Death Dis. 2024 Jul 5;15(7):484. doi: 10.1038/s41419-024-06870-9 (PMC11226677; doi:10.1038/s41419-024-06870-9)
Supplement: Supplementary file 1 — supplementary files including supplementary figures, supplementary table 1 and figure legends [file 41419_2024_6870_MOESM1_ESM.pdf]

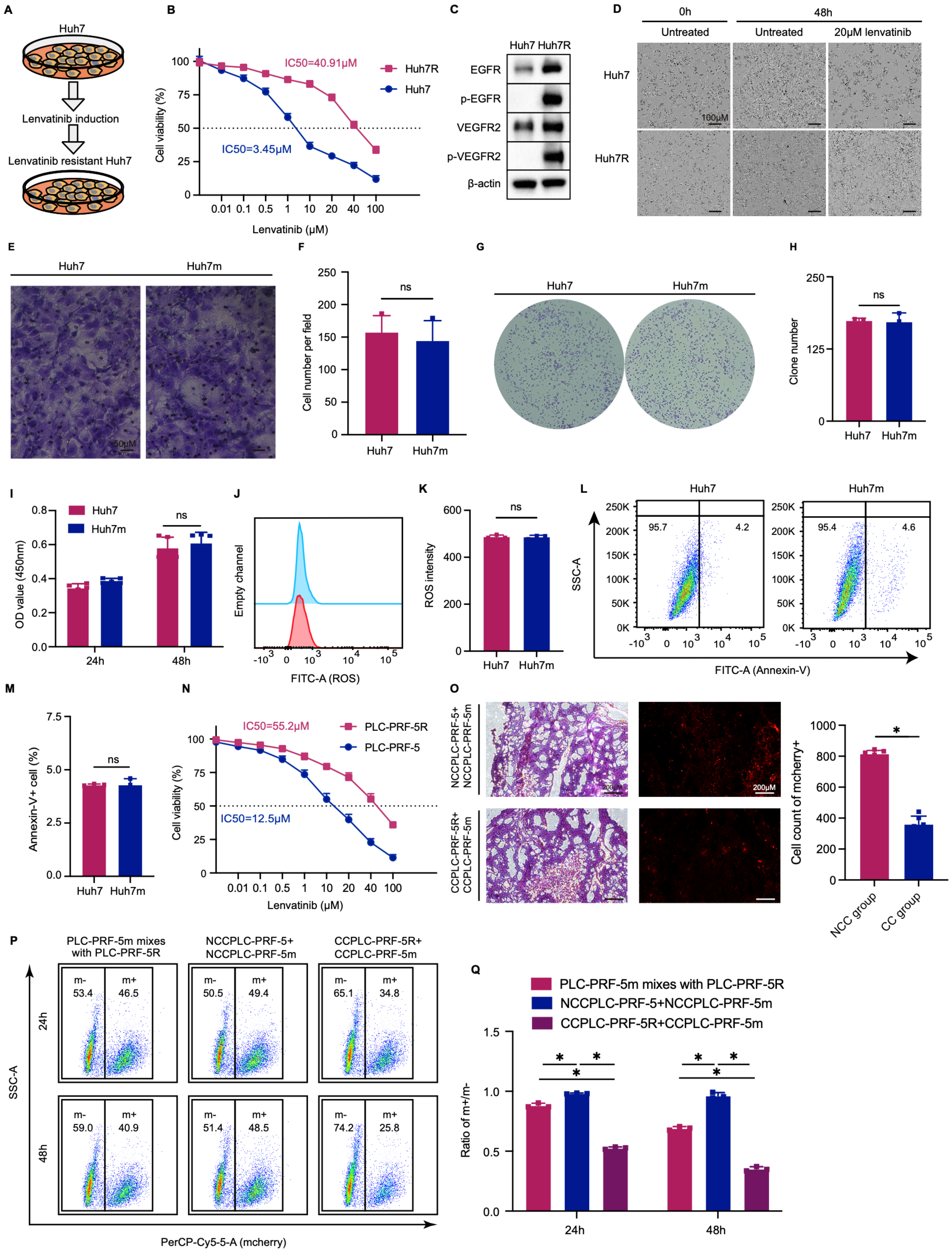

Supplementary Fig.1

Supplementary Figure 1. The validation of drug resistance of Huh7R and no influences of mcherry on cellular functions in Huh7.

(A) Schematic workflow of our experimental strategy. Huh7 was continuously cultured in gradually increasing concentrations of lenvatinib.

(B) Cell viability following varied concentrations of lenvatinib for 48h in Huh7 and Huh7R detected by CCK8.

(C) Protein levels of EGFR, p-EGFR, VEGFR2 and p-VEGFR2 in Huh7 and Huh7R detected by western blot.

(D) High content imaging of cells growth at 48h after cell attachment in Huh7 and Huh7R with or without 20 μm lenvatinib treatment using 10X objective lens.

(E) Microscope imaging of cell migration at 48h after cell attachment in Huh7 with or without mcherry transfection using 20X objective lens.

(F) Quantitative statistics of cell number in (E).

(G) Colony formation at 15 days after cell attachment in Huh7 and Huh7m.

(H) Quantitative statistics of clone number in (G).

(I) Quantified OD value of cell proliferation at 24h and 48h after cell attachment in Huh7 and Huh7m determined by CCK8.

(J) Flow cytometry analysis of ROS-FITC-A after cell attachment in Huh7 and Huh7m.

(K) Quantitative statistics of ROS-FITC-A in (J).

(L) Flow cytometry analysis of cell apoptosis Annexin-V-FITC-A after cell attachment in Huh7 and Huh7m.

(M) Quantitative statistics of early apoptosis Annexin-V-FITC-A in (L).

(N) (Left) Representative H&E stain and fluorescence imaging of cells in NCC group- and CC group-mice. (Right) Mcherry positive cells in NCC group and CC group were counted with ImageJ. N = 5.

(O) Flow cytometry analysis of cell proportion in NCC group, CC group and single PLC-PRF-5m mixed with single PLC-PRF-5R at 24h and 48h after cell attachment. mcherry+ (m+): PLC-PRF-5m, NCCPLC-PRF-5m, CC PLC-PRF-5m; mcherry- (m-): PLC-PRF-5R, NCCPLC-PRF-5, CC PLC-PRF-5R.

(P) Quantitative statiststics of ratio (m+/m-) in (O).

Three independent experiments were conducted, and the values are represented by means ± SEM using student's t test (F, H, I, K, M and N) or two-way ANOVA with Tukey's multiple comparisons test (P). *p < 0.05, ns = non-statistically significant.

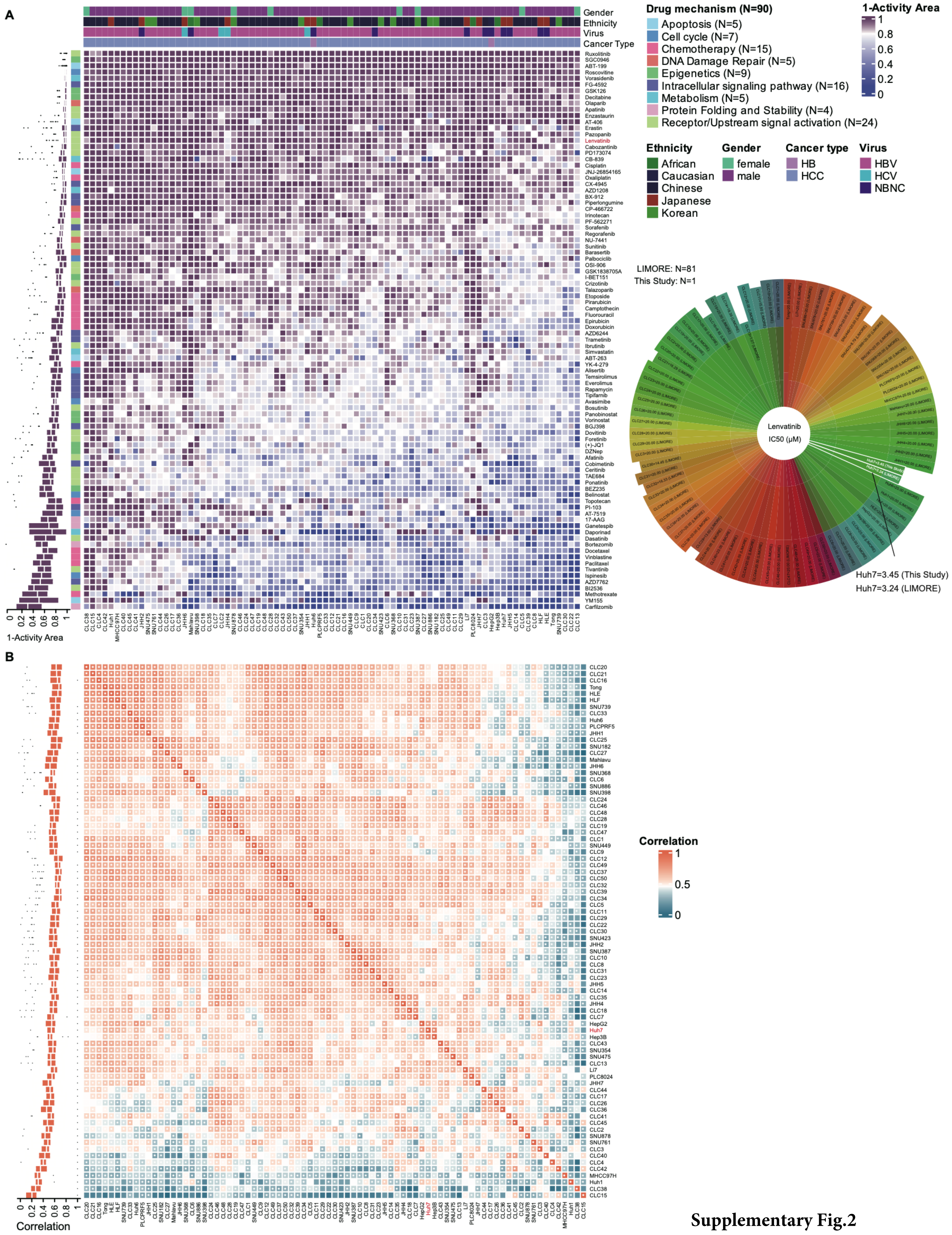

Supplementary Fig.2

Supplementary Figure S2. Common resistance pattern of lenvatinib and significant correlation in HCC cell lines.

(A) (Left) Box plot of drug response distributions and Heatmap plot of drug responses in 81 LIMORE HCC cell models. Drug response is displayed as the 1-Activity Area. (Right) Rose plot of IC50 of lenvatinib in LIMORE HCC cell models and Huh7 HCC cell (This Study).

(B) Heatmap plot of correlation in 81 LIMORE HCC cell models under lenvatinib treatment.

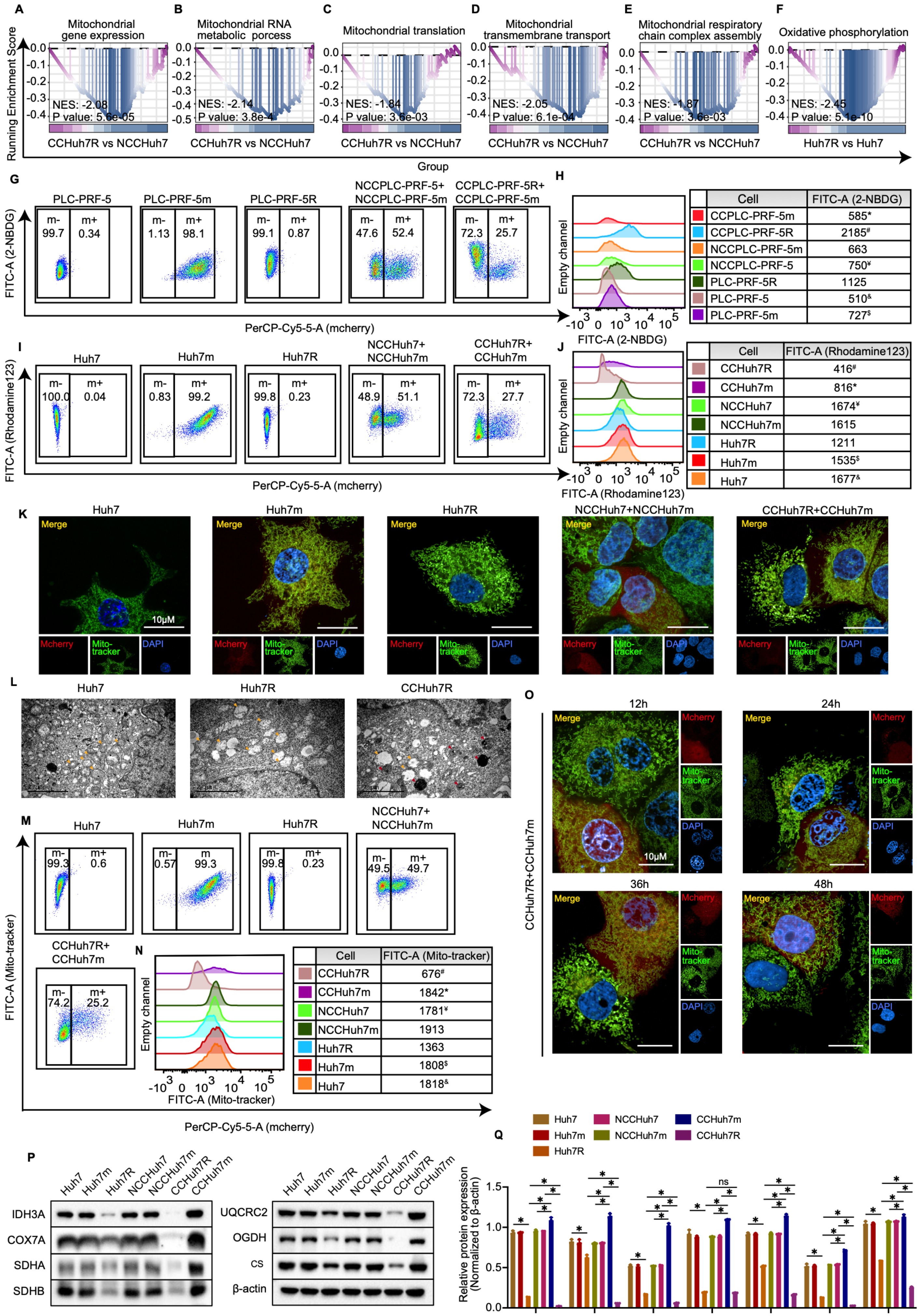

Supplementary Fig.3

Supplementary Figure S3. Cell competition attenuates oxidative phosphorylation of lenvatinib resistant cells in HCC.

(A) GSEA analysis of mitochondrial gene expression enrichment in CCHuh7R vs NCCHuh7 group.

(B) As in (A) but of mitochondrial RNA metabolic process enrichment.

(C) As in (A) but of mitochondrial translation enrichment.

(D) As in (A) but of mitochondrial transmembrane transport enrichment.

(E) As in (A) but of mitochondrial respiratory chain complex assembly enrichment.

(F) GSEA analysis of oxidative phosphorylation enrichment in Huh7R vs Huh7 group.

(G). Flow cytometry analysis of 2-NBDG-FITC-A and cell proportion at 48h after cell attachment in Alone group, NCC group and CC group.

(H) Mountain map of 2-NBDG-FITC-A in (J). * : CCPLC-PRF-5m vs NCCPLC-PRF-5m ($p < 0.05$); $: CCPLC-PRF-5m vs PLC-PRF-5m ($p < 0.05$); ¥ : CCPLC-PRF-5R vs NCCPLC-PRF-5 ($p < 0.05$); # : CCPLC-PRF-5R vs PLC-PRF-5R ($p < 0.05$); & : PLC-PRF-5R vs PLC-PRF-5 ($p < 0.05$).

(I) Flow cytometry analysis of Rhodamine123-FITC-A at 48h after cell attachment in Alone group, NCC group and CC group.

(J) Mountain map of Rhodamine123-FITC-A in (G). * : CCHuh7m vs NCCHuh7m ($p < 0.05$); $: CCHuh7m vs Huh7m ($p < 0.05$); ¥ : CCHuh7R vs NCCHuh7 ($p < 0.05$); # : CCHuh7R vs Huh7R ($p < 0.05$); & : Huh7R vs Huh7 ($p < 0.05$).

(K) High content fluorescence imaging of mitochondrial mass and morphology Mito-Tracker Green at 48h after cell adherence in Alone group, NCC group and CC group using 63X water immersion objective lens.

(L) Transmission electron microscope imaging of Huh7, Huh7R and CCHuh7R, the arrow (orange) indicates mitochondria of cell, the arrow (red) represents mitophagy activity in cell.

(M) Flow cytometry analysis of Mito-Tracker Green-FITC-A at 48h after cell adherence in Alone group, NCC group and CC group.

(N) Mountain map of Mito-Tracker Green-FITC-A in (J). * : CCHuh7m vs NCCHuh7m ($p < 0.05$); $: CCHuh7m vs Huh7m ($p < 0.05$); ¥ : CCHuh7R vs NCCHuh7 ($p < 0.05$); # : CCHuh7R vs Huh7R ($p < 0.05$); & : Huh7R vs Huh7 ($p < 0.05$).

(O) High content fluorescence imaging of Mito-Tracker Green at 12h, 24h, 36h and 48h after cell adherence in CC group using 63X water immersion objective lens.

(P) Protein levels of IDH3A, COX7A, SDHB, SDHA, UQCRC2, OGDH and CS at 48h after cell attachment in Alone group, NCC group and CC group detected by western blot.

(Q) Quantitative statistics of protein levels in (N).

Three independent experiments were conducted, and the values are represented by means ± SEM using two-way ANOVA with Turkey's multiple comparisons test. *$p < 0.05$, #$p < 0.05$, \$$p < 0.05$, ¥$p < 0.05$, &$p < 0.05$, ns = non-statistically significant.

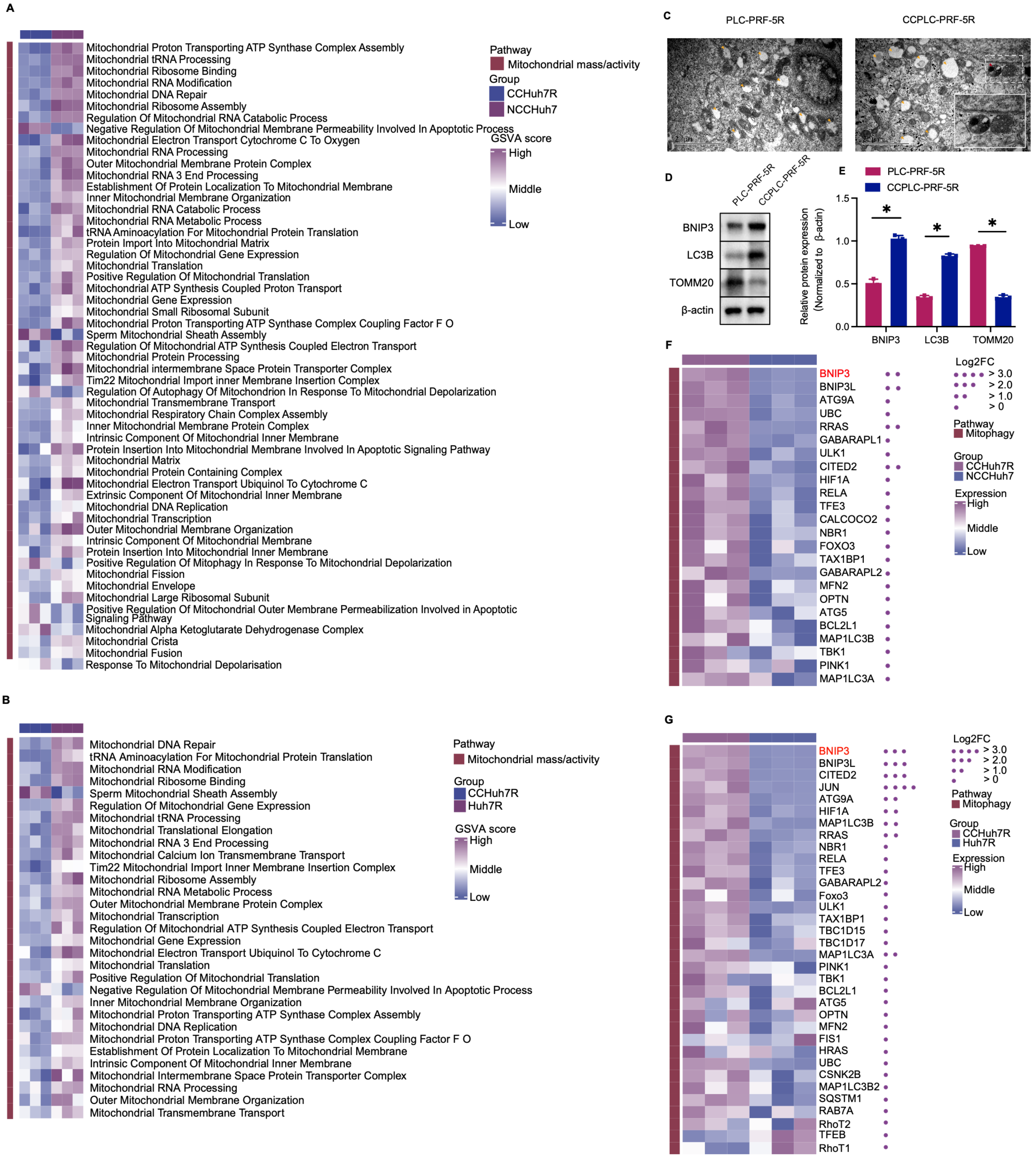

Supplementary Fig.4

Supplementary Figure S4. BNIP3-mediated mitophagy was observed and regulated energy metabolism reprogramming of lenvatinib-resistant cells.

(A) Heatmap showing the GSVA scores of related mitochondria mass or activities between CCHuh7R and NCCHuh7.

(B) As in (A) but between CCHuh7R and Huh7R.

(C) Transmission electron microscope imaging of PLC-PRF-5R and CCPLC-PRF-5R, the arrow (orange) indicates mitochondria of cell, the arrow (red) represents mitophagy activity in cell. The enlargement picture of the dashed boxes in CCPLC-PRF-5R shows the details of specific mitophagy activity.

(D) Protein levels of LC3B, TOMM20 and BNIP3 at 48h after cell attachment in PLC-PRF-5R and CCPLC-PRF-5R detected by western blot.

(E) Quantitative statistics of protein levels in (D).

(F) Heatmap exhibiting the expression levels of mitophagy-related genes in CCHuh7R vs NCCHuh7 group.

(G) As in (C) but in CCHuh7R vs Huh7R group.

Three independent experiments were conducted, and the values are represented by means ± SEM using Ordinary one-way ANOVA with Sidak's multiple comparisons test (E). $^*p < 0.05$, ns = non-statistically significant.

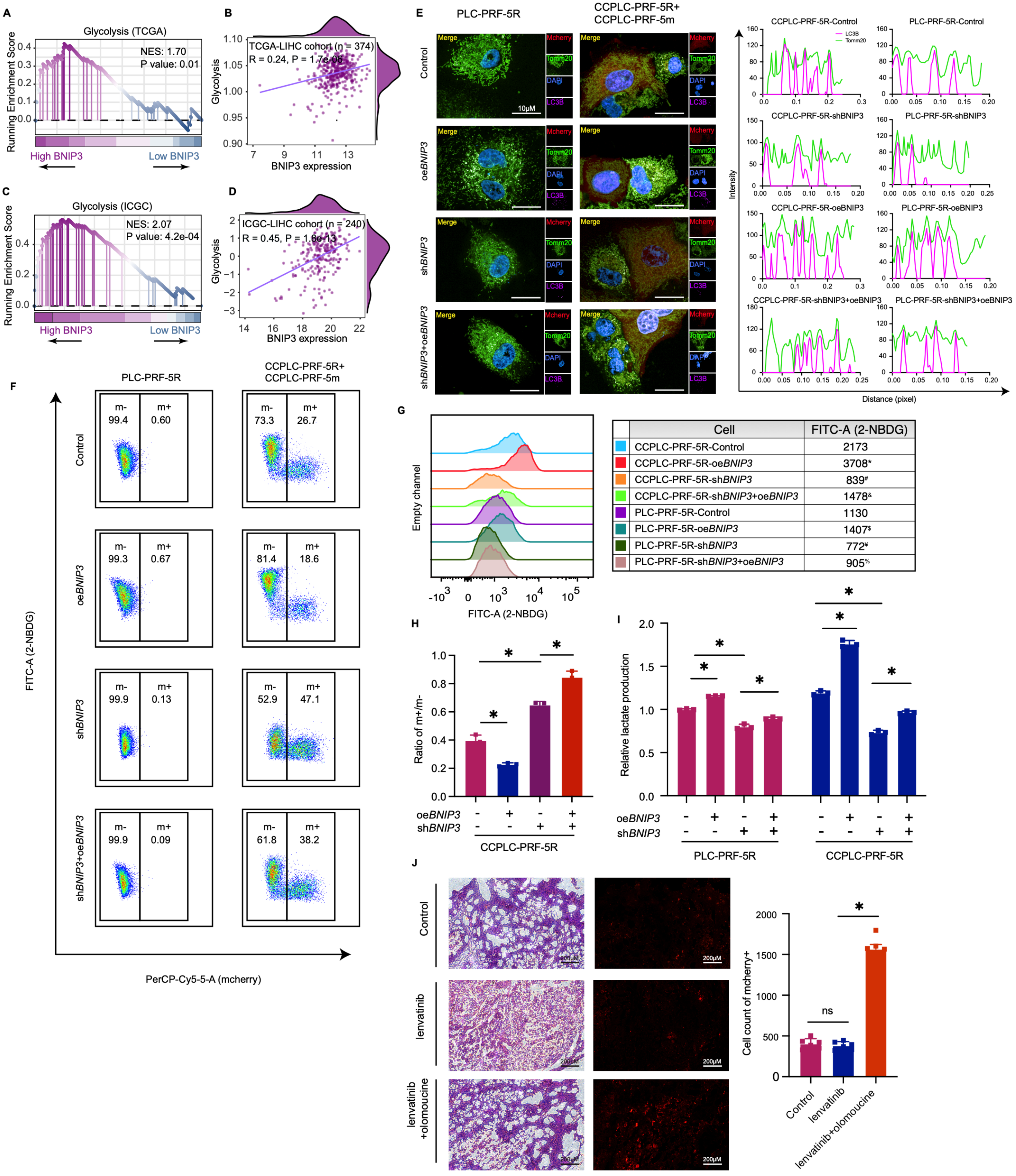

Supplementary Fig.5

Supplementary Figure S5 Mitophagy-dependent BNIP3 played a vital role in facilitating glycolysis metabolism in lenvatinib-resistant cells.

(A) GSEA analysis of glycolysis enrichment in BNIP3 high expression vs BNIP3 low expression group of TCGA-LIHC cohort.

(B) Correlation analysis of BNIP3 expression and glycolysis pathway in TCGA-LIHC cohort.

(C) GSEA analysis of glycolysis enrichment in BNIP3 high-expression vs BNIP3 low-expression group of ICGC-LIHC cohort.

(D) Correlation analysis of BNIP3 expression and glycolysis pathway in ICGC-LIHC cohort.

(E) (Left) High content immunofluorescence imaging of colocalization of autophagosomes (Cy5.5-LC3: purple) and mitochondria (TOMM20: green) at 48h after cell attachment in PLC-PRF-5R (Alone group) /CCPLC-PRF-5R (CC group) transfected with shRNA against *BNIP3* (sh*BNIP3*), *BNIP3* overexpression plasmid (oe*BNIP3*) or oe*BNIP3*+sh*BNIP3* using 63X water immersion objective lens. (Right) Quantification of colocalization between LC3 (purple peak) and TOMM20 (green peak) in the above groups. Purple/green peak height represents fluorescence intensity of LC3B/TOMM20; overlapping peaks indicate colocalization numbers between LC3B and Tomm20.

(F) Flow cytometry analysis of 2-NBDG-FITC-A and cell proportion at 48h after cell attachment in PLC-PRF-5R (Alone group) /CCPLC-PRF-5R (CC group) transfected with sh*BNIP3*, oe*BNIP3* or sh*BNIP3*+oe*BNIP3.*

(G) Mountain map of 2-NBDG-FITC-A in (F). * : CCPLC-PRF-5R -oe*BNIP3* vs CCPLC-PRF-5R-Control ($p < 0.05$); # : CCPLC-PRF-5R-sh*BNIP3* vs CCPLC-PRF-5R-Control (p

< 0.05); & : CCPLC-PRF-5R-sh*BNIP3*+oe*BNIP3* vs CCPLC-PRF-5R-sh*BNIP3* (p < 0.05); $: PLC-PRF-5R-oe*BNIP3* vs PLC-PRF-5R-Control (p < 0.05); ¥ : PLC-PRF-5R-sh*BNIP3* vs PLC-PRF-5R-Control (p < 0.05); % : PLC-PRF-5R-sh*BNIP3*+oe*BNIP3* vs PLC-PRF-5R-sh*BNIP3* (p < 0.05).

(H) Quantitative statistics of ratio m+/m- in (F).

(I) Cellular lactic acid production levels at 48h after cell attachment in PLC-PRF-5R (Alone group) /CCPLC-PRF-5R (CC group) transfected with sh*BNIP3*, oe*BNIP3* or sh*BNIP3*+oe*BNIP3* by flow cytometry cell sorting.

(J) (Left) Representative H&E stain and fluorescence imaging of cells in CC group-mice with or without lenvatinib or lenvatinib+olomouine. (Right) Mcherry positive cells in corrresponding groups were counted with ImageJ. N = 5.

Three independent experiments were conducted, and the values are represented by means ± SEM using two-way ANOVA with multiple comparisons test (I) or Ordinary one-way ANOVA with Sidak’s multiple comparisons test (H and J). *p < 0.05, #p < 0.05, $p < 0.05, ¥p < 0.05, &p <0.05, %p < 0.05, ns = non-statistically significant.

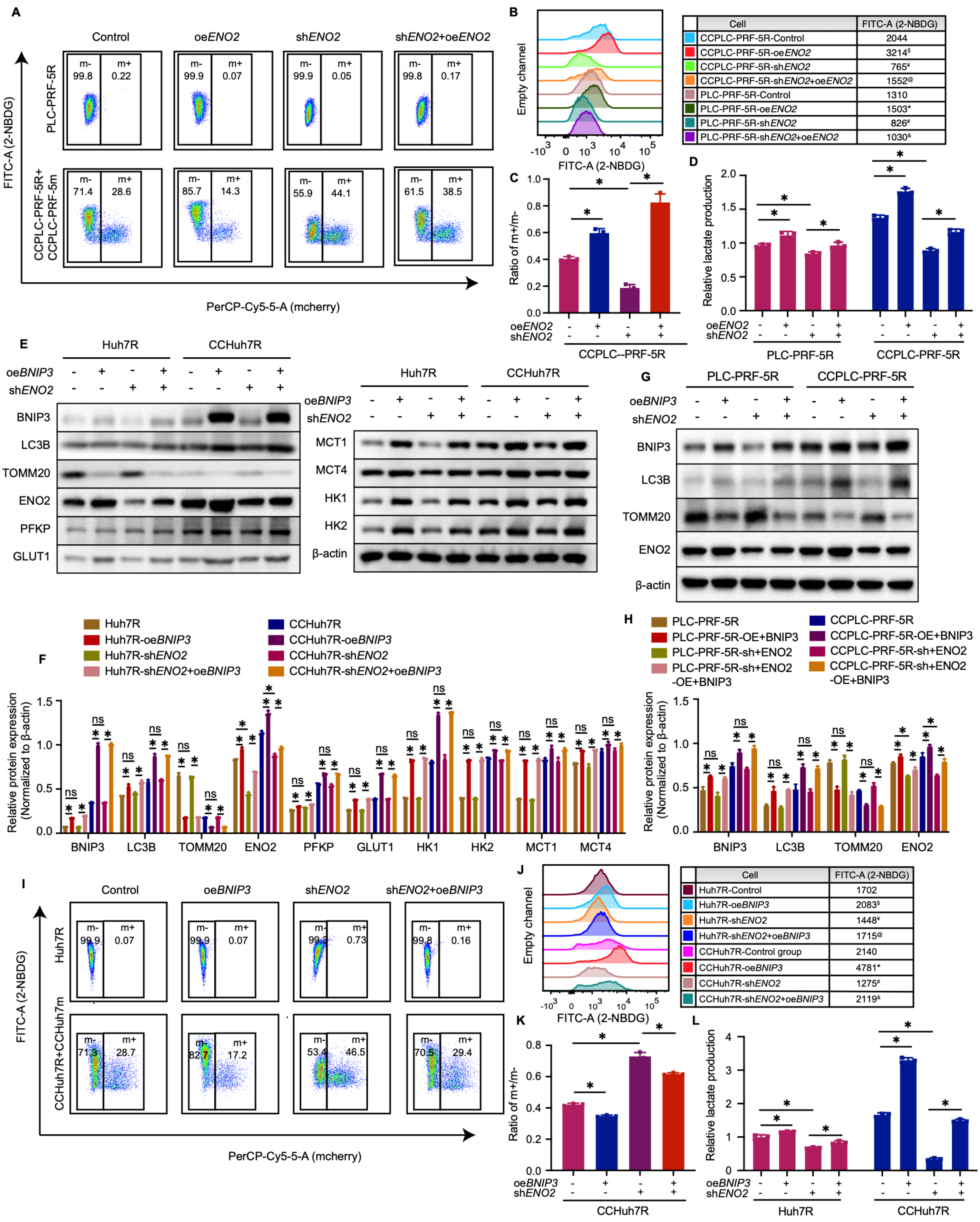

Supplementary Fig.6

Supplementary Figure S6. BNIP3 mediated mitophagy regulated glycolysis by ENO2 to support the winner status of lenvatinib-resistant cells.

(A) Flow cytometry analysis of 2-NBDG-FITC-A and cell proportion at 48h after cell attachment in PLC-PRF-5R (Alone group) /CCPLC-PRF-5R (CC group) transfected with shRNA against *ENO2* (sh*ENO2*), *ENO2* overexpression plasmid (oe*ENO2*) or oe*ENO2*+sh*ENO2*.

(B) Mountain map of 2-NBDG-FITC-A in (D). * : CCPLC-PRF-5R-oe*ENO2* vs CCPLC-PRF-5R-Control ($p < 0.05$); # : CCPLC-PRF-5R-sh*ENO2* vs CCPLC-PRF-5R-Control ($p < 0.05$); & : CCPLC-PRF-5R-sh*ENO2+*oe*ENO2* vs CCPLC-PRF-5R-sh*ENO2* ($p < 0.05$); $: PLC-PRF-5R-oe*ENO2* vs PLC-PRF-5R-Control ($p < 0.05$); ¥ : PLC-PRF-5R-sh*ENO2* vs PLC-PRF-5R-Control ($p < 0.05$); % : PLC-PRF-5R-sh*ENO2+*oe*ENO2* vs PLC-PRF-5R-sh*ENO2* ($p < 0.05$).

(C) Quantitative statistics of ratio m+/m- in (A).

(D) Cellular lactic acid production levels at 48h after cell attachment in PLC-PRF-5R (Alone group) /CCPLC-PRF-5R (CC group) transfected with sh*ENO2*, oe*ENO2* or sh*ENO2*+oe*ENO2* by flow cytometry cell sorting.

(E) Protein levels of LC3B, TOMM20, BNIP3, ENO2, GLUT1, HK1, HK2, MCT1, MCT4 and PFKP at 48h after cell attachment in Huh7R (Alone group) /CCHuh7R (CC group) transfected with sh*ENO2*, oe*BNIP3* or sh*ENO2*+oe*BNIP3* with detected by western blot.

(F) Quantitative statistics of protein levels in (E).

(G) Protein levels of LC3B, TOMM20, BNIP3, ENO2 at 48h after cell attachment in PLC-PRF-5R (Alone group) /CCPLC-PRF-5R (CC group) transfected with sh*ENO2*, oe*BNIP3* or sh*ENO2*+oe*BNIP3* with detected by western blot.

(H) Quantitative statistics of protein levels in (G).

(I) Flow cytometry analysis of 2-NBDG-FITC-A and cell proportion at 48h after cell attachment in Huh7R (Alone group) /CCHuh7R (CC group) transfected with sh*ENO2*, oe*BNIP3* or sh*ENO2*+oe*BNIP3.*

(J) Mountain map of 2-NBDG-FITC-A in (G). * : CCHuh7R-oe*BNIP3* vs CCHuh7R-Control ($p < 0.05$); # : CCHuh7R-sh*ENO2* vs CCHuh7R-Control ($p < 0.05$); & : CCHuh7R-sh*ENO2*+oe*BNIP3* vs CCHuh7R- sh*ENO2* ($p < 0.05$); $: Huh7R-oe*BNIP3* vs Huh7R-Control ($p < 0.05$); ¥ : Huh7R-sh*ENO2* vs Huh7R-Control ($p < 0.05$); % : Huh7R-sh*ENO2*+oe*BNIP3* vs Huh7R-sh*ENO2* ($p < 0.05$).

(K) Quantitative statistics of ratio m+/m- in (I).

(L) Lactic acid production levels at 48h after cell attachment in Huh7R (Alone group) /CCHuh7R (CC group) transfected with sh*ENO2*, oe*BNIP3* or sh*ENO2*+oe*BNIP3* by flow cytometry cell sorting.

Three independent experiments were conducted, and the values are represented by means ± SEM using a two-way ANOVA with Turkey's multiple comparisons test (D, F, H and L) or Ordinary one-way ANOVA with Sidak's multiple comparisons test (C, K). *p < 0.05, #p < 0.05, $p < 0.05, ¥p < 0.05, &p <0.05, %p < 0.05, ns = non-statistically significant.

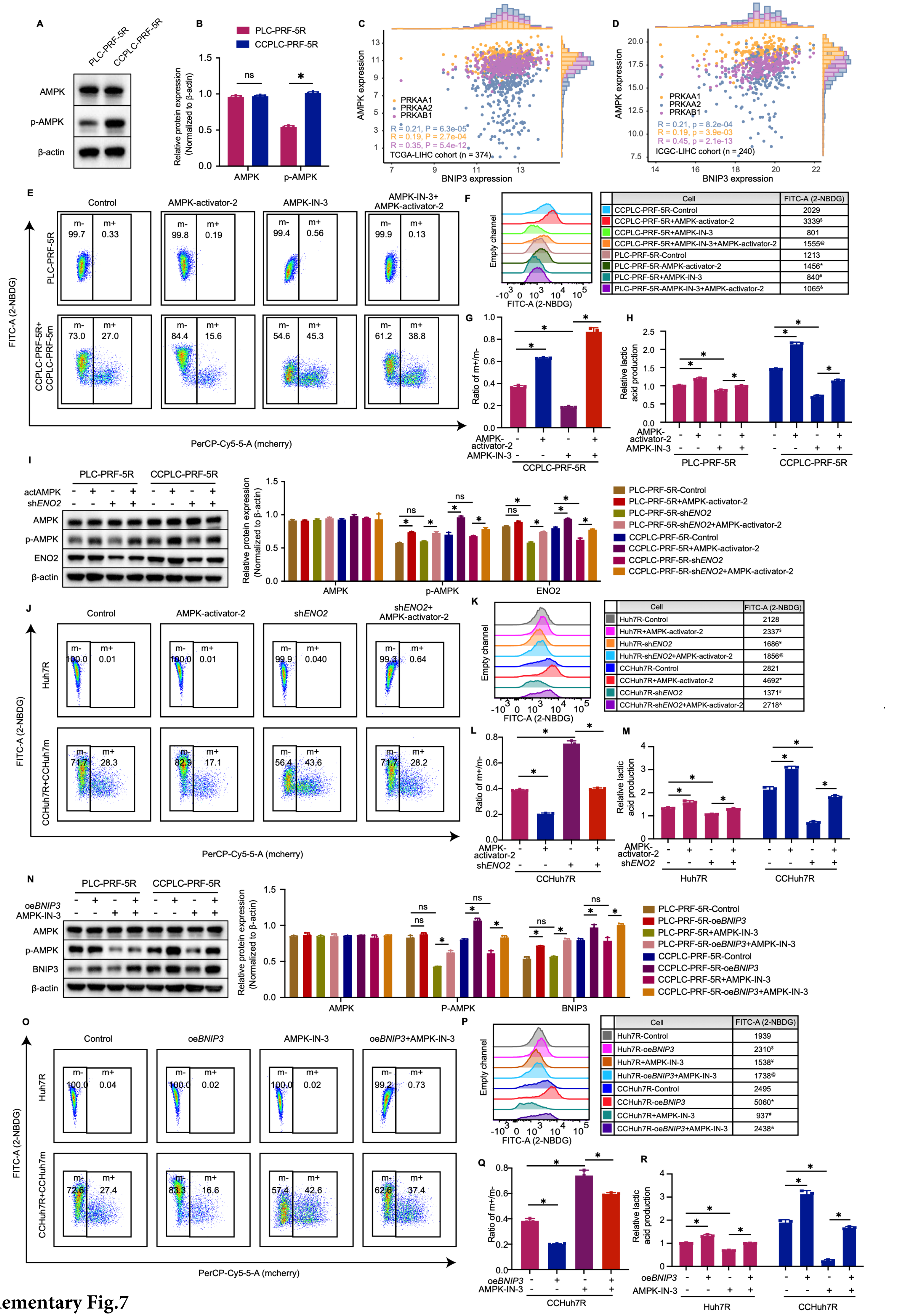

Supplementary Fig.7

Supplementary Figure S7. BNIP3-AMPK-ENO2 axis mediated the process of energy metabolism shifting of lenvatinib-resistant cells in cell competition.

(A) Protein levels of AMPK and p-AMPK at 48h after cell attachment in PLC-PRF-5R and CCPLC-PRF-5R detected by western blot.

(B) Quantitative statistics of protein levels in (A).

(C) Correlation analysis of BNIP3 expression and AMPK expression in TCGA-LIHC cohort.

(D) As in (A) but in ICGC-LIHC cohort.

(E) Flow cytometry analysis of 2-NBDG-FITC-A and cell proportion at 48h after cell attachment in PLC-PRF-5R (Alone group) /CCPLC-PRF-5R (CC group) treated with AMPK-activator-2, AMPK-IN-3 or AMPK-IN-3+AMPK-activator-2.

(F) Mountain map of 2-NBDG-FITC-A in (G). * : CC PLC-PRF-5R+AMPK-activator-2 vs CCPLC-PRF-5R-Control ($p < 0.05$); # : CCPLC-PRF-5R-AMPK-IN-3 vs CCPLC-PRF-5R-Control ($p < 0.05$); & : CCPLC-PRF-5R+AMPK-IN-3+AMPK-activator-2 vs CCPLC-PRF-5R+AMPK-IN-3 ($p < 0.05$); $: PLC-PRF-5R-AMPK-activator-2 vs PLC-PRF-5R-Control ($p < 0.05$); ¥ : PLC-PRF-5R+AMPK-IN-3 vs PLC-PRF-5R-Control ($p < 0.05$); % : PLC-PRF-5R+AMPK-IN-3+AMPK-activator-2 vs PLC-PRF-5R+AMPK-IN-3 ($p < 0.05$).

(G) Quantitative statistics of ratio m+/m- in (E).

(H) Cellular lactic acid production levels at 48h after cell attachment in PLC-PRF-5R (Alone group) /CCPLC-PRF-5R (CC group) treated with AMPK-activator-2, AMPK-IN-3 or AMPK-IN-3+AMPK-activator-2 by flow cytometry cell sorting.

(I) (Left) Protein levels of AMPK, p-AMPK and ENO2 at 48h after cell attachment in PLC-PRF-5R (Alone group) /CCPLC-PRF-5R (CC group) treated with AMPK-activator-

2, sh*ENO2* or sh*ENO2*+AMPK-activator-2 detected by western blot. (Right) Quantitative statistics of protein levels.

(J) Flow cytometry analysis of 2-NBDG-FITC-A and cell proportion at 48h after cell attachment in Huh7R (Alone group) /CCHuh7R (CC group) treated with AMPK-activator-2, sh*ENO2* or sh*ENO2*+AMPK-activator-2.

(K) Mountain map of 2-NBDG-FITC-A in (C). * : CCHuh7R+AMPK-activator-2 vs CCHuh7R-Control ($p < 0.05$); # : CCHuh7R-sh*ENO2* vs CCHuh7R-Control ($p < 0.05$); & : CCHuh7R-sh*ENO2*+AMPK-activator-2 vs CCHuh7R-sh*ENO2* ($p < 0.05$); $: Huh7R+AMPK-activator-2 vs Huh7R-Control ($p < 0.05$); ¥ : Huh7R-sh*ENO2* vs Huh7R-Control ($p < 0.05$); % : Huh7R-sh*ENO2*+AMPK-activator-2 vs Huh7R-sh*ENO2* ($p < 0.05$).

(L) Quantitative statistics of ratio m+/m- in (J).

(M) Lactic acid production levels at 48h after cell attachment in Huh7R (Alone group) /CCHuh7R (CC group) treated with AMPK-activator-2, sh*ENO2* or sh*ENO2*+AMPK-activator-2 by flow cytometry cell sorting.

(N) (Left) Protein levels of AMPK, p-AMPK and BNIP3 at 48h after cell attachment in PLC-PRF-5R (Alone group) /CCPLC-PRF-5R (CC group) treated with oe*BNIP3*, AMPK-IN-3 or oe*BNIP3*+AMPK-IN-3 detected by western blot. (Right) Quantitative statistics of protein levels.

(O) Flow cytometry analysis of 2-NBDG-FITC-A and cell proportion at 48h after cell attachment in Huh7R (Alone group) /CCHuh7R (CC group) treated with oe*BNIP3*, AMPK-IN-3 or oe*BNIP3*+AMPK-IN-3.

(P) Mountain map of 2-NBDG-FITC-A in (F). * : CCHuh7R-oe*BNIP3* vs CCHuh7R-Control (p < 0.05); # : CCHuh7R+AMPK-IN-3 vs CCHuh7R-Control (p < 0.05); & : CCHuh7R-oe*BNIP3*+AMPK-IN-3 vs CCHuh7R+AMPK-IN-3 (p < 0.05); $: Huh7R-oe*BNIP3* vs Huh7R-Control (p < 0.05); ¥ : Huh7R+AMPK-IN-3 vs Huh7R-Control (p < 0.05); % : Huh7R-oe*BNIP3*+AMPK-IN-3 vs Huh7R+AMPK-IN-3 (p < 0.05).

(Q) Quantitative statistics of ratio m+/m- in (O).

(R) Lactic acid production levels at 48h after cell attachment in Huh7R (Alone group) /CCHuh7R (CC group) treated with oe*BNIP3*, AMPK-IN-3 or oe*BNIP3*+AMPK-IN-3 by flow cytometry cell sorting.

Three independent experiments were conducted, and the values are represented by means ± SEM using a two-way ANOVA with Turkey's multiple comparisons test (H, I, M, N and R) or Ordinary one-way ANOVA with Sidak's multiple comparisons test (D, G, L and Q). *p < 0.05, #p < 0.05, $p < 0.05, ¥p < 0.05, &p <0.05, %p < 0.05, ns = non-statistically significant.

Supplementary Video.

Video 1. High content imaging of Huh7 cultured alone for 48h in the absence of lenvatinib using 10X objective lens.

Video 2. As in Video1 but in the presence of 20μm lenvatinib.

Video 3. As in Video1 but of Huh7R.

Video 4. As in Video3 but in the presence of 20μm lenvatinib.

Video 5. High content imaging of Huh7R cocultured with Huh7m at 1:1 ratio for 48h using 20X objective lens.

Video 6. High content imaging of Huh7 cultured alone for 48h using 10X objective lens.

Video 7. As in Video 6 but of Huh7m.

Video 8. As in Video 6 but of Huh7R.

Video 9. High content imaging of Huh7 cocultured with Huh7m at 1:1 ratio for 48h using 10X objective lens.

Video 10. High content imaging of Huh7R cocultured with Huh7m at 1:1 ratio for 48h using 10X objective lens.

Video 11. High content imaging of Huh7R cocultured with Huh7m at 1:1 ratio for 48h using 10X objective lens.

Video 12. High content imaging of Huh7R cocultured with Huh7m at 1:1 ratio for 48h under low glucose treatment using 10X objective lens.

Video 13. As in Video 12 but under Calcitriol treatment.

Three independent experiments were conducted.

Table 1. Sequences for plasmid or shRNA.

**Recombinant DNA**	**Source**	**Sequence**
BNIP3 overexpression plasmid (Human) Forward:	Sangon	ATGTCGCAGAACGGAGCGCCCG
BNIP3 overexpression plasmid (Human) Reverse:	Sangon	TCAAAAGGTGCTGGTGGAGGTT
ENO2 overexpression plasmid (Human) Forward:	Sangon	ATGTCCATAGAGAAGATCTGGG
ENO2 overexpression plasmid (Human) Reverse:	Sangon	TCACAGCACACTGGGATTACGG
**Oligonucleotides**	**Source**	**Sequence**
BNIP3 shRNA#1: Sense:	Liver Cancer Institute	GCCACGTCACTTGTGTTTATT
BNIP3 shRNA#2: Sense:	Liver Cancer Institute	GCTTCTGAAACAGATACCCAT
BNIP3 shRNA#3: Sense:	Liver Cancer Institute	GCTCTCTCATTTGCTGGCCAT
ENO2 shRNA#1: Sense:	Liver Cancer Institute	CGCCTGGCTAATAAGGCTTTA
ENO2 shRNA#2: Sense:	Liver Cancer Institute	GCCGGACATAACTTCCGTAAT
ENO2 shRNA#3: Sense:	Liver Cancer Institute	CATCAAGGACAAATACGGCAA
ENO2 shRNA#4: Sense:	Liver Cancer Institute	CGCACTTTCCACTTCTTCCTT
ENO2 shRNA#5: Sense:	Liver Cancer Institute	CGTTCTGAACGTCTGGCTAAA
