# Supplementary material for: BNIP3-mediated mitophagy boosts the competitive growth of Lenvatinib-resistant cells via energy metabolism reprogramming in HCC
Source: Cell Death Dis. 2024 Jul 5;15(7):484. doi: 10.1038/s41419-024-06870-9 (PMC11226677; doi:10.1038/s41419-024-06870-9)

Supplemental Figure 8

Full and Uncropped western blot for Supplemental Fig. S1C

EGFR

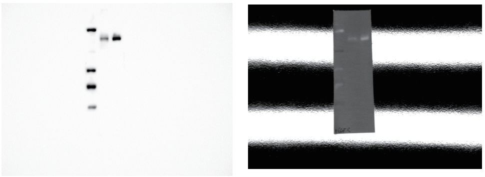

p-EGFR

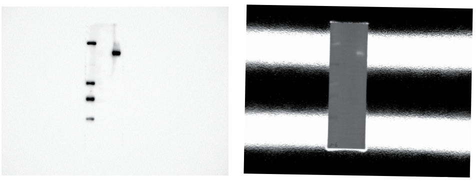

VEGFR2

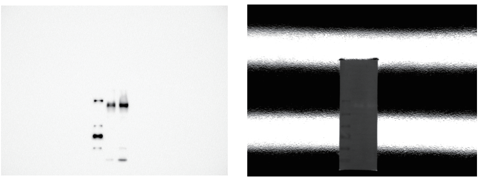

p-VEGFR2

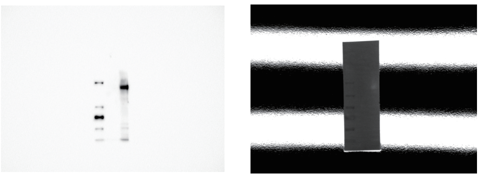

β-actin

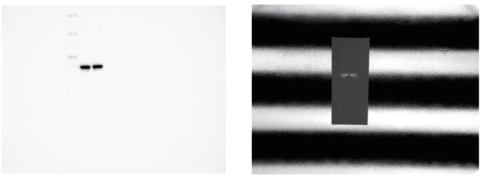

Full and Uncropped western blot for Fig. 2M

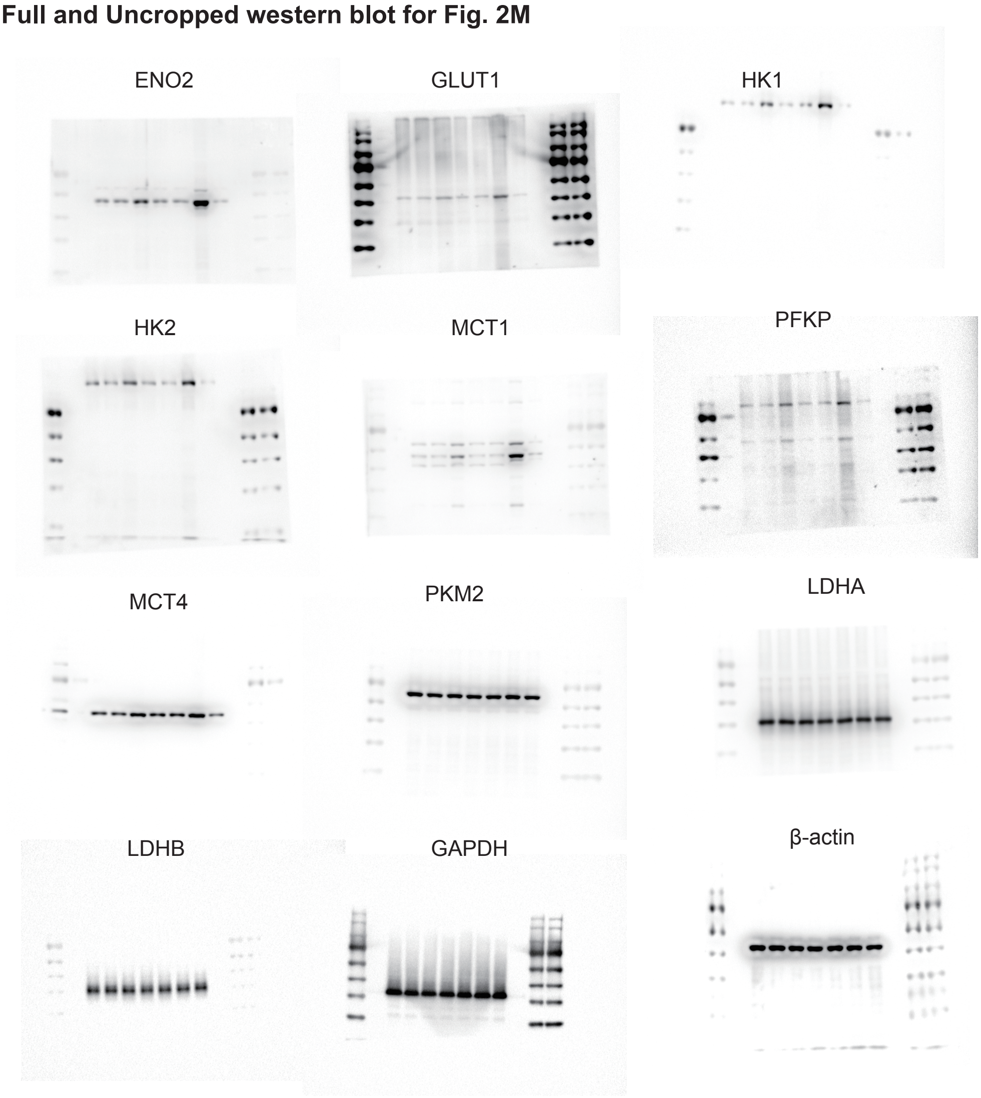

Full and Uncropped western blot for Supplemental Fig. S3M

IDH3A

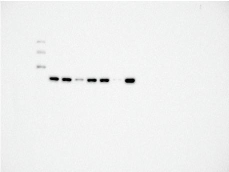

IDH3A_UV

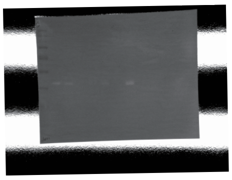

COX7A

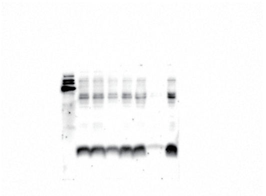

COX7A_UV

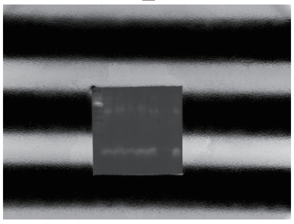

SDHA

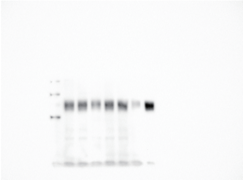

SDHA_UV

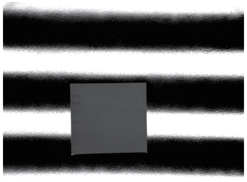

SDHB

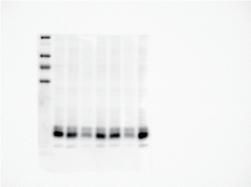

SDHB_UV

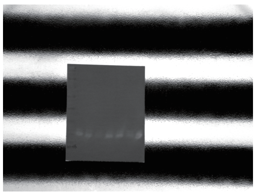

UQCRC2

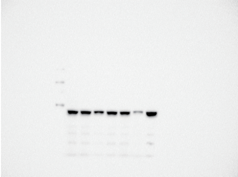

UQCRC2_UV

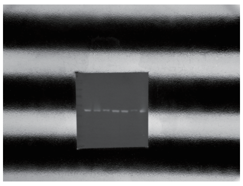

CS

CS_UV

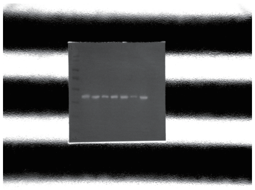

OGDH

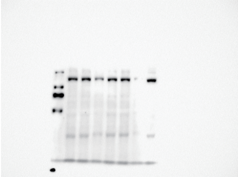

OGDH_UV

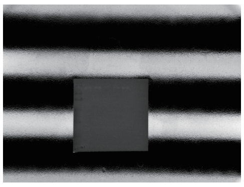

β-actin

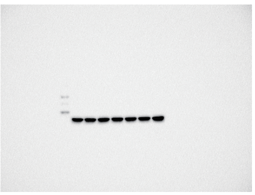

β-actin_UV

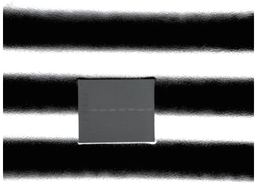

Full and Uncropped western blot for Fig. 4C

BNIP3

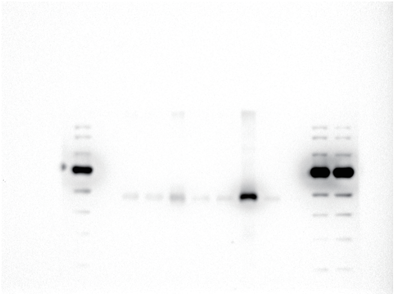

TOMM20

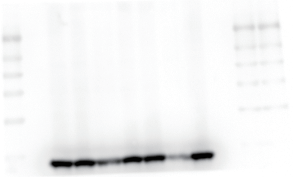

LC3B

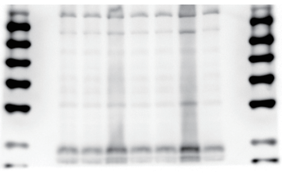

β-actin

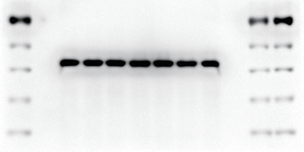

Full and Uncropped western blot for Fig. 4F

BNIP3

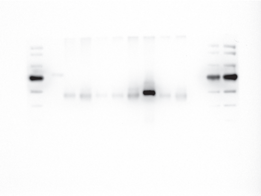

LC3B

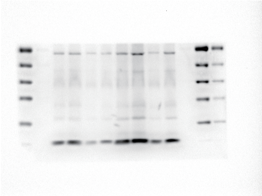

TOMM20

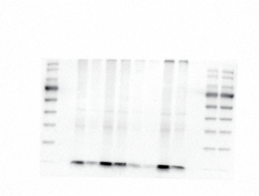

PFKP

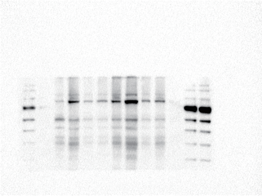

ENO2

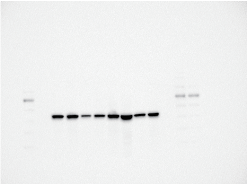

GLUT1

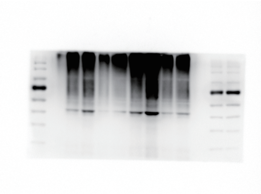

MCT1

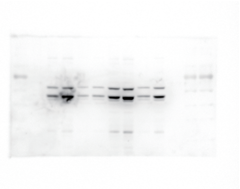

MCT4

HK1

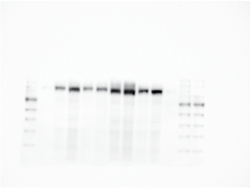

HK2

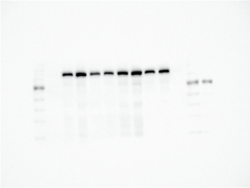

β-actin

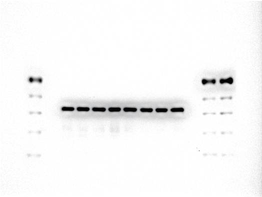

Full and Uncropped western blot for Fig. 5H

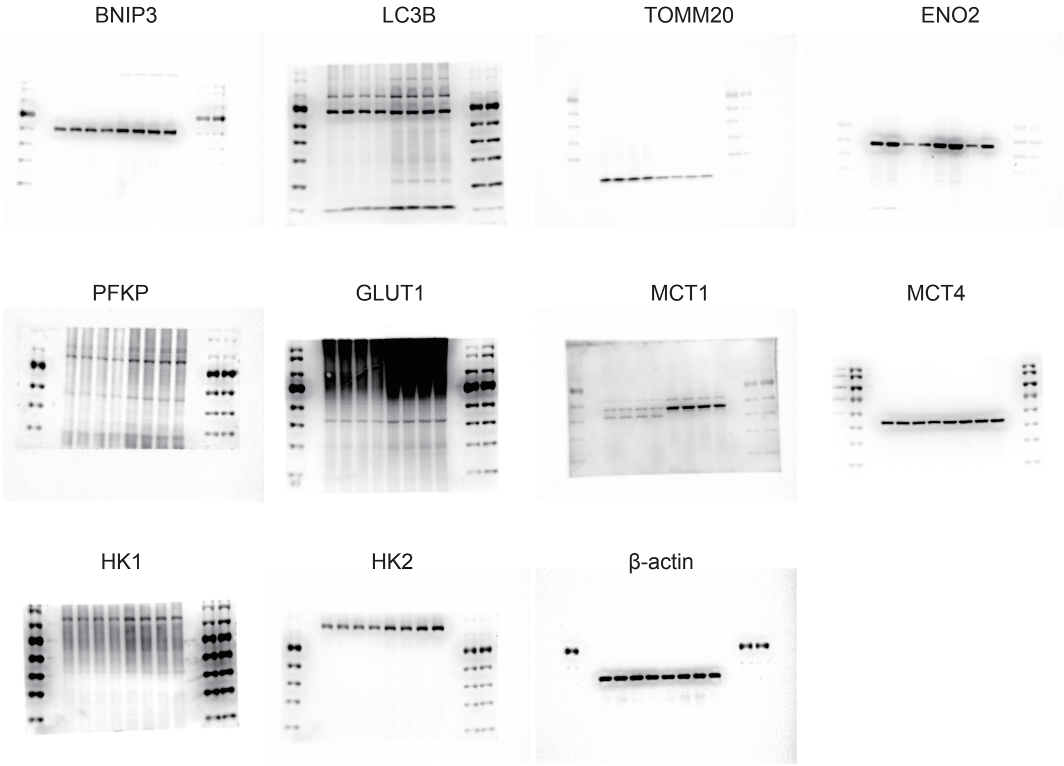

Full and Uncropped western blot for Fig. Supplemental Fig. S6E

BNIP3

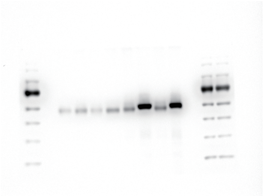

LC3B

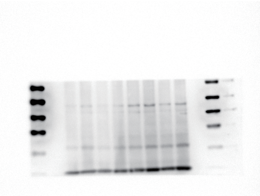

TOMM20

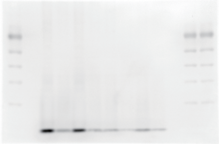

ENO2

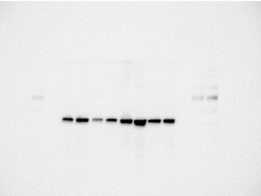

PFKP

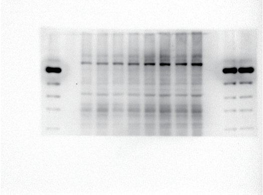

GLUT1

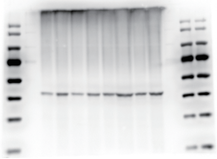

MCT1

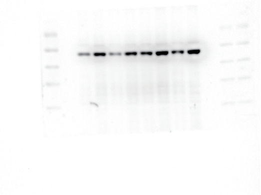

MCT4

HK1

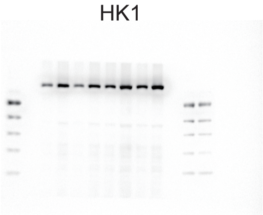

HK2

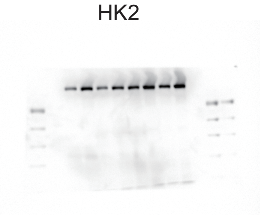

β-actin

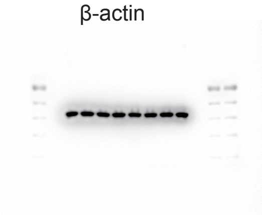

Full and Uncropped western blot for Fig. Supplemental Fig. S6G

BNIP3

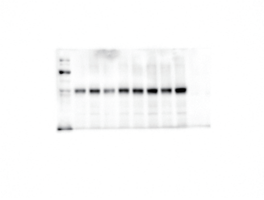

BNIP3-UV

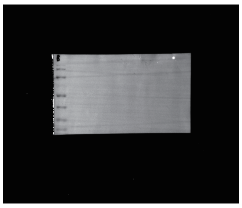

LC3B

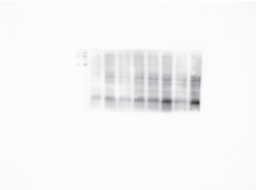

LC3B-UV

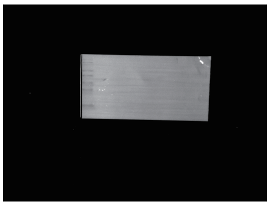

TOMM20

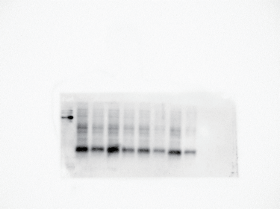

TOMM20-UV

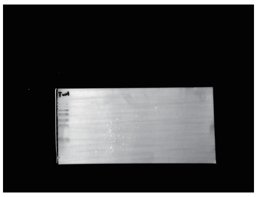

ENO2

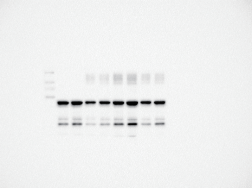

ENO2-UV

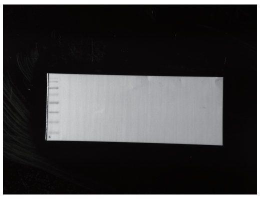

β-actin

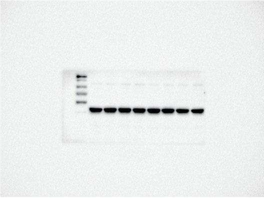

Full and Uncropped western blot for Fig. 6C

AMPK

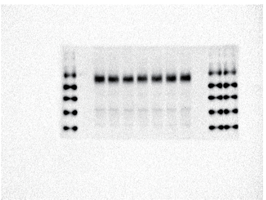

p-AMPK

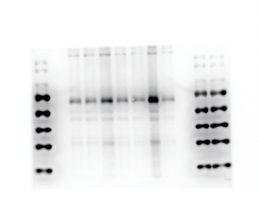

β-actin

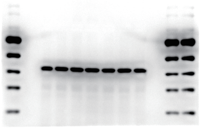

Full and Uncropped western blot for Fig. 6L

AMPK

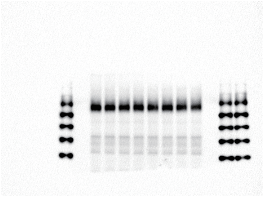

p-AMPK

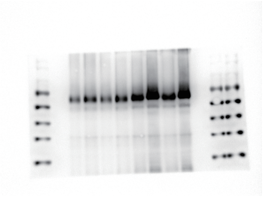

ENO2

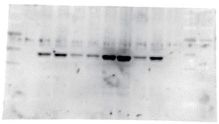

β-actin

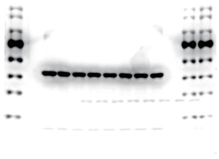

Full and Uncropped western blot for Fig. 6N

AMPK

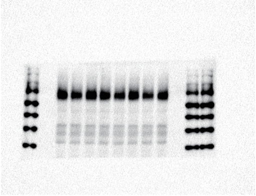

p-AMPK

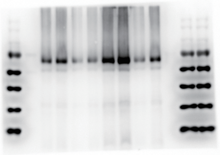

BNIP3

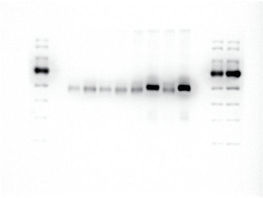

β-actin

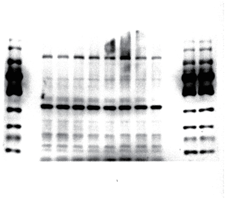

Full and Uncropped western blot for Fig. Supplemental Fig. S7A

AMPK

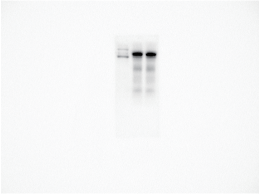

AMPK-UV

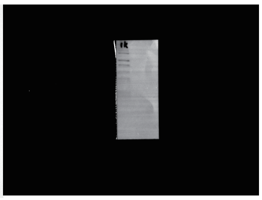

p-AMPK

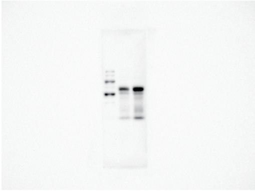

p-AMPK-UV

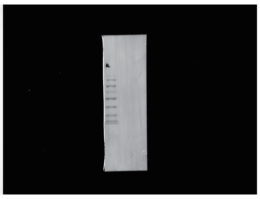

β-actin

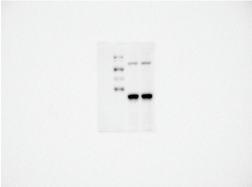

Full and Uncropped western blot for Fig. Supplemental Fig. S7I

AMPK

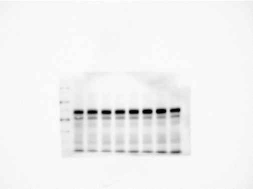

AMPK-UV

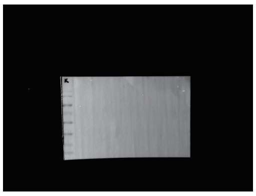

p-AMPK

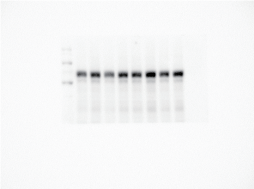

p-AMPK-UV

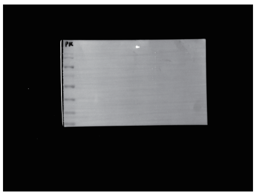

ENO2

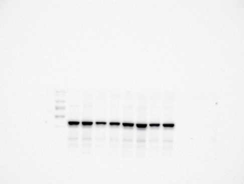

ENO2-UV

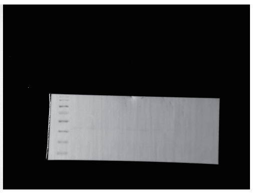

β-actin

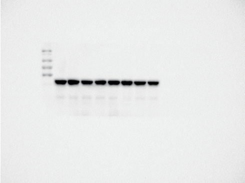

Full and Uncropped western blot for Fig. Supplemental Fig. S7N

AMPK

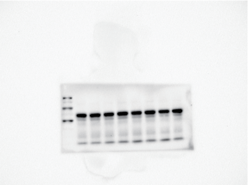

AMPK-UV

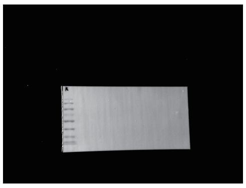

p-AMPK

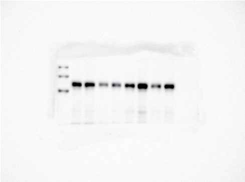

p-AMPK-UV

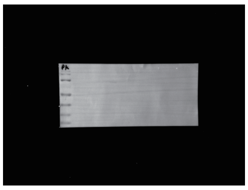

BNIP3

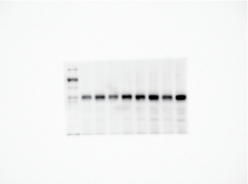

BNIP3-UV

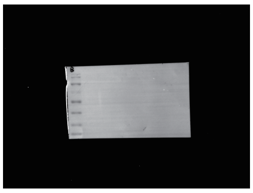

β-actin

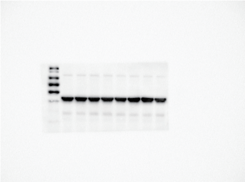

Supplement: Supplementary file 15 — full and uncropped WB images [file 41419_2024_6870_MOESM15_ESM.pdf]
